# Disability in long-term care residents explained by prevalent geriatric syndromes, not long-term care home characteristics: a cross-sectional study

**DOI:** 10.1186/s12877-017-0444-1

**Published:** 2017-02-10

**Authors:** Natasha E. Lane, Walter P. Wodchis, Cynthia M. Boyd, Thérèse A. Stukel

**Affiliations:** 1 Institute of Health Policy, Management and Evaluation, University of Toronto, 155 College St, 4th Floor, Toronto, ON M5T 3M6 Canada; 2 Institute for Clinical Evaluative Sciences, G1 06 – 2075 Bayview Avenue, Toronto, ON M4N 3M5 Canada; 3 Toronto Rehabilitation Institute, 550 University Avenue, 3rd Floor, Toronto, ON M5G 2A2 Canada; 4 Johns Hopkins School of Medicine, 1830 E. Monument St, Baltimore, MD 21287 USA; 5 Johns Hopkins Bloomberg School of Public Health, 615 North Wolfe Street, Baltimore, MD 21205 USA; 6 Johns Hopkins Center on Aging and Health, 2024 E. Monument St, Suite 2-700, Baltimore, MD 21205 USA; 7 Dartmouth Institute for Health Policy & Clinical Practice, Geisel School of Medicine at Dartmouth, Hanover, NH 03755 USA

**Keywords:** Activities of daily living, Chronic disease, Disability, Disablement Process, Geriatric syndrome, Nursing homes

## Abstract

**Background:**

Self-care disability is dependence on others to conduct activities of daily living, such as bathing, eating and dressing. Among long-term care residents, self-care disability lowers quality of life and increases health care costs. Understanding the correlates of self-care disability in this population is critical to guide clinical care and ongoing research in Geriatrics. This study examines which resident geriatric syndromes and chronic conditions are associated with residents’ self-care disability and whether these relationships vary across strata of age, sex and cognitive status. It also describes the proportion of variance in residents’ self-care disability that is explained by residents’ geriatric syndromes versus long-term care home characteristics.

**Methods:**

We conducted a cross-sectional study using a health administrative cohort of 77,165 long-term care home residents residing in 614 Ontario long-term care homes. Eligible residents had their self-care disability assessed using the RAI-MDS 2.0 activities of daily living long-form score (range: 0–28) within 90 days of April 1st, 2011. Hierarchical multivariable regression models with random effects for long-term care homes were used to estimate the association between self-care disability and resident geriatric syndromes, chronic conditions and long-term care home characteristics. Differences in findings across strata of sex, age and cognitive status (cognitively intact versus cognitively impaired) were examined.

**Results:**

Geriatric syndromes were much more strongly associated with self-care disability than chronic conditions in multivariable models. The direction and size of some of these effects were different for cognitively impaired versus cognitively intact residents. Residents’ geriatric syndromes explained 50% of the variation in their self-care disability scores, while characteristics of long-term care homes explained an additional 2% of variation.

**Conclusion:**

Differences in long-term care residents’ self-care disability are largely explained by prevalent geriatric syndromes. After adjusting for resident characteristics, there is little variation in self-care disability associated with long-term care home characteristics. This suggests that residents’ geriatric syndromes—not the homes in which they live—may be the appropriate target of interventions to reduce self-care disability, and that such interventions may need to differ for cognitively impaired versus unimpaired residents.

**Electronic supplementary material:**

The online version of this article (doi:10.1186/s12877-017-0444-1) contains supplementary material, which is available to authorized users.

## Background

Long-term care homes (LTCHs) are publicly-funded facilities for older adults whose care needs are greater than the level provided by home care or retirement homes, but less than that provided in hospital [1]. Demand for institutional long-term care is increasing globally, as are the acuity and complexity of LTCH (or “nursing home”) residents [2]. Most LTCH residents have some self-care disability, defined as difficulty with or dependence on others to conduct activities of daily living (ADLs), such as bathing, eating and dressing [1]. Self-care disability (henceforth “disability”) tends to increase over time among LTCH residents [3] and is associated with lower self-rated quality of life [4], repeat hospitalizations [5], higher health care utilization [6] and all-cause mortality [7, 8]. Based on its association with these important resident outcomes, resident disability measures are included in pay-for-performance schemes and publicly reported LTCH quality metrics in jurisdictions across North America [9–11].

There is limited evidence regarding the association of specific resident and LTCH characteristics with resident disability [12], or the extent that these associations differ by age [13], sex [14] and cognitive status [15]. Identifying the resident or LTCH characteristics that explain differences in resident disability could guide targeting of clinical interventions to prevent or slow its onset. Determining whether the effects of geriatric syndromes and chronic conditions on disability differ by age, sex and cognitive status is important because imbalanced effect modifiers in research samples skew findings. Existing studies of these relationships are limited by small or single-sex samples, inadequate control for confounders, lack of adjustment for clustering of residents within LTCHs, and selection bias due to voluntary LTCH participation [16–19].

We conducted a cross-sectional administrative data study to answer the following questions and fill these evidence gaps:

1. Which resident geriatric syndromes and chronic conditions are most strongly associated with disability in LTCH residents?
2. Are these relationships moderated by residents’ sex, age or cognitive status?
3. What is the proportion of variance in resident disability explained by resident characteristics versus LTCH characteristics?

Our examination of these questions was guided by Verbrugge and Jette’s Disablement Process Model [20]. The Disablement Process Model is a theoretical framework that outlines a pathway through which pathologies lead to impairments, which give way to limitations in functional capacity [20]. Reduced functional capacity then interacts with individuals’ sociodemographic characteristics and context to cause disability [20]. Additional file 1: Table S1 contains definitions and examples for pathology, impairment, functional limitation, sociodemographic (“intra-individual”) characteristics and contextual (“extra-individual”) factors from Verbrugge and Jette’s Disablement Process Model paper [20]. There was an additional “risk factors” construct in the original Disablement Process Model, however because all of the risk factors were also sociodemographic characteristics these categories were collapsed in this study.

We incorporated this framework into this study in two ways. First, our variable selection and model specification were based on constructs in the Disablement Process Model. The “chronic pathologies” construct was represented by chronic conditions – such as heart failure or Parkinson’s disease—defined as “illnesses lasting six months or more, including past illnesses requiring continuous care, diseases with risk of recurrence or previous health problems that continue to affect health management” [21]. The “impairments” construct was represented by geriatric syndromes,—such as balance impairment or urinary incontinence—defined as “a collection of signs and symptoms common in older residents but not necessarily fitting into discrete disease categories” [22]. Second, we tested an extension of the original model to see whether there is effect modification of exposure-disability relationships by resident age, sex and cognitive status. We hypothesized that the effects of chronic diseases and geriatric syndromes on disability would be stronger among women, the oldest old and individuals who were cognitively impaired.

## Methods

**Fig. 1:**
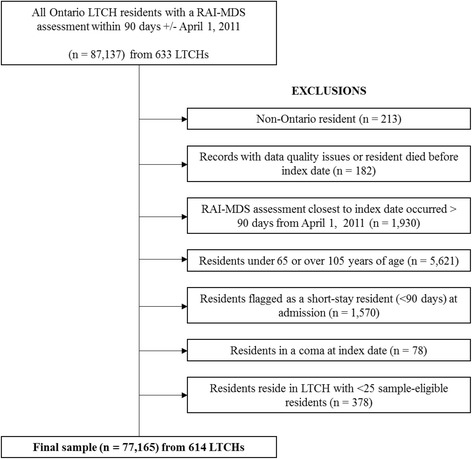
(Study cohort creation for all Ontario LTCH residents with a RAI-MDS assessment within 90 days +/− April 1, 2011)

We enrolled all LTCH residents in Ontario, Canada, whose disability was assessed within 90 days (+/−) of the index date, April 1, 2011. We then applied several exclusions (Fig. 1) and used residents’ de-identified and encrypted provincial health insurance numbers to link health administrative databases housed at the Institute for Clinical Evaluative Sciences (ICES).

### Data sources

Resident records were linked using unique, anonymized, encrypted identifiers across multiple Ontario health administrative databases containing information on all publicly insured, medically necessary hospital and physician services. These included the Discharge Abstract Database (DAD) for chronic conditions coded during hospital admissions; the Ontario Health Insurance Plan (OHIP) for physician billings, including diagnosis codes and procedures; the Registered Persons Database (RPDB) for resident age and sex; and the Continuing Care Reporting System (CCRS) which includes administrative information on LTCH characteristics and patient-level data from the Resident Assessment Instrument Minimum Dataset 2.0 (RAI-MDS) assessments carried out in LTCHs [23]. The RAI-MDS is a standardized, multidimensional assessment tool used in LTCHs across Canada, the US and Europe [24]; this study used Ontario RAI-MDS data on resident disability, demographic characteristics, and chronic condition and geriatric syndrome diagnoses. Trained LTCH staff completed the assessments when residents were admitted to LTCH, quarterly, and when there was any significant resident health status change [25].

### Outcome

The primary outcome was resident disability, measured using the ADL long-form score (ADL LFS) from the RAI-MDS assessment closest to the index date. The ADL LFS quantifies resident disability from 0 to 28 based on degree of dependence on others for bed mobility, transfer, locomotion, dressing, eating, toilet use and personal hygiene (see Additional file 1: Table S2). Higher values of ADL LFS indicate higher disability. The ADL LFS is less prone to ceiling effects than more abbreviated disability scales [26], has been validated against standardized measures of disability [27, 28], and is reliable [29]. Although it is an ordinal measure, it was treated as a continuous variable in this study, in keeping with statistical guidelines [30] and precedent in other research [16, 31–33].

### Exposures

Prevalent geriatric syndromes and chronic conditions were the primary exposures of interest. The accrual period for chronic condition diagnoses was five years prior to the index date. Chronic conditions were coded as prevalent if they were identified in hospital or physician billing data as primary or comorbid diagnoses in one inpatient or two outpatient visits within two years of each other [34] or if they were denoted as “active conditions” in RAI-MDS assessments at least once. Geriatric syndromes were coded as present as indicated in residents’ RAI-MDS assessment closest to the index date. The full set of 16 chronic conditions and nine geriatric syndromes included, as well as the diagnostic codes used to define them, are listed in Additional file 1: Tables S3 and S4.

### Covariates

Selection of resident and LTCH-level covariates for multivariable models was guided by the Disablement Process Model [20]. Resident-level covariates included age, sex, marital status, pre-admission neighborhood income quintile and the number of days since admission to the LTCH. LTCH-level variables based on aggregate resident characteristics (e.g. proportion of residents restrained) were calculated using all residents in each LTCH who were assessed within 90 days (+/−) of the index date and were still alive on the index date.

### Statistical analyses

The frequency and distribution of resident and LTCH characteristics in the sample were determined. Bivariate unadjusted relationships between resident and LTCH characteristics and disability were assessed in linear regression models. A null model containing only random LTCH intercepts and no other explanatory variables was run and a likelihood ratio test determined whether there was significant between-LTCH variance in resident disability. Hierarchical multivariable Model 1 contained only random LTCH effects and resident variables, whereas Model 2 contained random LTCH effects, resident and LTCH variables. This model sequence facilitated stepwise calculation of the total proportion of variance in disability explained by variables in each model (*R* ^*2*^), and the proportion of variance in disability that was between LTCHs in the sample (ρ) [35]. To test for multicollinearity of the variables in the multivariable model, the variance inflation factor (VIF) was estimated for each predictor in the reported model (Model 1). The assumption of normally distributed residual errors was also verified. To test whether the effect of chronic diseases and geriatric syndromes on disability were stronger among women, the oldest residents and individuals who were cognitively impaired, we conducted a descriptive analysis of Model 1 stratified by sex, age and cognitive status (cognitively intact or borderline versus cognitive impaired [36]).

#### Sensitivity analyses

We re-ran Model 1 with fixed effects instead of random effects for LTCHs to examine whether unmeasured LTCH effects were biasing our findings. Fixed effects account for individual LTCH’s effect on variance in residents’ disability, without specifying the LTCH variables responsible, whereas random effects adjust for the overall variation across all LTCHs. If coefficient estimates from the fixed effects version of Model 1 differed significantly from the random effects version, it would suggest that our estimation was biased by relevant LTCH characteristics that we were unable to measure. A linear regression with no random or fixed effects for LTCHs was also run. We also re-ran Model 1, alternatively removing all geriatric syndromes, all chronic conditions, and all variables except for four geriatric syndromes to test how sensitive effects for each type of exposure was to adjustment for the other. To examine the sensitivity of our findings to coding of chronic conditions, we re-ran Model 1 using chronic condition codes from claims data only, then using chronic condition codes from RAI-MDS data only. Model 2 was re-run excluding residents whose data were from admission assessments to examine whether their inclusion weakened relationships between LTCH characteristics and resident disability. Descriptive analyses were done using SAS version 9.3 [37] and regression modelling was done in STATA.

## Results

### Resident and long-term care home characteristics

A total of 77,165 residents from 614 LTCHs were included in the sample and are described in Table 1. The median disability score for all residents in the sample was 18 (IQR: 9, 23); 71.2% of them were female and their mean age was 84.9 years (SD: 7.5). LTCHs had an average of 126 (SD: 67.3) active beds and the majority of homes in the sample were classified as medium size, for-profit, and located in urban settings (Table 2). There was very little variation in LTCH-level mean disability associated with different levels of the measured LTCH characteristics (Table 2).

**Table 1:** Characteristics of long-term care residents in sample

Characteristics	N	%	Mean Disability (SD)
Full Cohort	77,165	100	16.1 (8.4)
Age (years)			
65–74	7,859	10.2	15.1 (8.0)^‡^
75–84	25,703	33.3	15.9 (8.6)^‡^
85–94	36,676	47.5	16.2 (8.3)^‡^
95+	6,927	9.0	17.6 (7.7)^‡^
Sex			
Female	54,953	71.2	16.4 (8.4)^‡^
Male	22,212	28.8	15.4 (8.5)^‡^
Marital Status			
Married	18,632	17.0	17.0 (8.4)^‡^
Widowed	46,067	16.1	16.1(8.4)^‡^
Never married/Separated/Divorced	11,299	14.8	14.8 (8.6)^‡^
Missing data	1,167	15.7	15.7 (8.4)^‡^
Pre-LTCH Neighborhood Income Quintile			
1 (low)	17,671	22.9	15.4 (8.5)^‡^
2	13,510	17.5	16.0 (8.4)^‡^
3	13,473	17.5	15.9 (8.5)^‡^
4	11,790	15.3	16.4 (8.3)^‡^
5 (high)	10,909	14.1	16.4 (8.4)^‡^
Missing data	9,812	12.7	17.0 (8.4)^‡^
Days in LTCH Prior to Index Date			
0–4 months	19,202	24.9	15.3 (8.1)^‡^
> 4 months - 12 months	14,045	18.2	15.0 (8.2)^‡^
> 1 year - 2 years	13,854	17.9	15.3 (8.4)^‡^
> 2 years - 3 years	8,515	11.0	16.1 (8.5)^‡^
> 3 years	21,549	27.9	18.0 (8.6)^‡^
Prevalent Geriatric Syndromes			
Balance impairment	59,502	77.1	18.5 (7.4)^‡^
Bowel incontinence	37,966	49.2	21.4 (5.7)^‡^
Cognitive status			
Intact or borderline intact	18,426	23.9	10.9 (8.0)^‡^
Mild/moderate impairment	37,204	48.2	14.8 (7.5)^‡^
Moderate-severe/severe impairment	21,535	27.9	22.7 (5.8)^‡^
Hearing impairment			
None	66,718	86.5	15.9 (8.5)^‡^
Hearing impaired	10,269	13.3	17.6 (8.0)^‡^
Missing data	178	0.2	15.3 (8.3)^‡^
Body mass index (BMI)			
BMI < 18.5	6683	8.7	18.7 (7.8)^‡^
18.5 ≤ BMI ≤ 25	32,614	42.3	16.7 (8.4)^‡^
25 < BMI <30	22,134	28.7	15.2 (8.6)^‡^
BMI ≥ 30	15,734	20.4	15.0 (8.3)^‡^
Pain			
None	46,595	60.4	16.3 (8.5)^‡^
Less than daily pain	17,895	23.2	15.6 (8.2)^‡^
Daily or severe daily pain	12,675	16.4	16.2 (8.4)^‡^
Pressure ulcer	4,834	6.3	22.2 (6.0)^‡^
Urinary incontinence	54,922	71.2	19.1 (6.8)^‡^
Visual impairment			
None	43,701	56.6	14.4 (8.4)^‡^
Moderate impairment	27,264	35.3	17.5 (7.9)^‡^
Severe impairment	6,022	7.8	22.0 (7.2)^‡^
Missing	178	0.2	15.3 (8.3)^‡^
Prevalent Chronic Conditions			
Arthritis	48,114	62.4	15.8 (8.3)^‡^
Asthma	5,740	7.4	15.4 (8.3)^‡^
Cancer	25,016	32.4	15.3 (8.4)^‡^
Kidney disease	17,124	22.2	16.1 (8.2)
Coronary artery disease	29,999	38.9	15.6 (8.4)^‡^
Chronic obstructive pulmonary disease	16,823	21.8	15.0 (8.3)^‡^
Dementia	65,291	84.6	16.6 (8.3)^‡^
Diabetes	24,456	31.7	16.0 (8.3)
Epilepsy	5,262	6.8	18.1 (8.3)^‡^
Heart failure	19,430	25.2	15.9 (8.1)^†^
Limb paralysis or amputation	7,031	9.1	20.2 (6.5)^‡^
Mood disorders	32,389	42.0	16.4 (8.3)^‡^
Parkinson’s disease	7,082	9.2	18.7 (7.6)^‡^
Peripheral vascular disease	7,132	9.2	16.0 (8.1)
Psychiatric conditions other than depression and dementia	21,288	27.6	15.1 (8.5)^‡^
Stroke	17,005	22.0	17.4 (7.9)^‡^

All *p*-values from ANOVAs to test differences in ADL LFS across different levels of each category

^†^ *p*-value <0.01

^‡^ *p*-value <0.0001

**Table 2:** Characteristics of long-term care homes (LTCHs) in sample

Characteristic	N	%	Grand Mean (SD) of homes’ ADL LFS means
Full Sample	614	100	15.7 (2.0)
LTCH Size (# beds)			
Small (≤64)	128	20.8	15.4 (2.3)
Medium (65–128)	248	40.4	15.5 (2.2)
Large (129–192)	154	25.1	15.7 (2.2)
Extra-large (≥193)	84	13.7	16.0 (2.0)
Ownership status			
Not-for-profit	228	37.1	15.5 (2.2)^*^
For-profit	378	61.6	15.8 (2.1)^*^
Missing data	8	1.3	12.1 (2.4)^*^
Location			
Rural	136	22.2	14.8 (2.1)^*^
Sub-urban (census agglomerations)	97	15.8	14.9 (1.92)^*^
Urban (census metropolitan areas)	381	62.1	16.0 (2.1)^*^
Receipt of Rehabilitation Services			
Lowest quartile (Received by ≤74.5% of residents in home)	153	24.9	15.3 (2.2)^*^
2nd quartile (Received by >74.5% and ≤86.4% of residents in home)	154	25.1	15.3 (2.3)^*^
3rd quartile (Received by >86.4, and ≤94.1% of residents in home)	154	25.1	15.9 (2.2)^*^
Highest quartile (Received by >94.7% of residents in home)	153	24.9	15.8 (2.0)^*^
Restraint use			
Lowest quartile (Homes in which ≤6.0% residents restrained)	153	24.9	15.3 (2.6)^*^
2nd quartile (Homes in which >6.0% and ≤13.4% residents restrained)	154	25.1	15.2 (2.0)^*^
3rd quartile (Homes in which >13.4% and ≤20.6% residents restrained)	153	24.9	15.5 (2.1)^*^
Highest quartile (Homes in which ≥20.6% residents restrained)	154	25.1	16.4 (1.8)^*^
Median ADL of residents in each home^§^			
Lowest quartile (Homes whose residents’ median ADL LFS ≤15)	176	28.7	13.2 (1.3)^*^
2nd quartile (Homes whose residents median ADL LFS >15, ≤ 17)	180	29.3	15.3 (0.9)^*^
3rd quartile (Homes whose residents median ADL LFS >17, < 19)	103	16.8	16.2 (0.9)^*^
Highest quartile (Homes whose residents ADL LFS ≥19)	155	25.2	18.2 (1.3)^*^

^*^Significant (*p <*0.05) difference between levels of categorical variable according to ANOVA

^§^The 614 LTCHs in the sample did not divide into quartiles of even size because of the small range of values for this variable and large number of homes with identical values

### Multivariable models of disability in long-term care residents

The coefficients in Table 3 represent the association of chronic conditions and geriatric syndromes with the 29-point ADL LFS measure of disability. Variables with significant positive coefficients (e.g. Parkinson’s) are associated with greater disability, whereas variables with significant negative coefficients (e.g. coronary artery disease) are associated with less disability, adjusting for other variables in the table. A one-point increase in ADL LFS is considered the minimum threshold for clinical significance, as it indicates increased dependence in an ADL or dependence in a new ADL, both of which are associated with intensified care needs from LTCH staff [38]. Because LTCH characteristics had small and non-significant effects on disability, estimates from Model 1—which includes random effects for LTCHs but no LTCH characteristics—are reported. Coefficient estimates for all covariates included in Models 1 and 2 can be found in Additional file 1: Table S5. The mean VIF for variables in Model 1 is 1.53 (Range: 1.04–3.69), which falls far below the threshold (VIF ≥ 10) indicative of multicollinearity.

**Table 3:** Geriatric syndromes and chronic conditions associated with disability in long-term care residents

	Unadjusted Bivariate Regressions	Model 1^§^
	Estimate (95% CI)	Estimate (95% CI)
Prevalent Geriatric Syndromes		
Balance impairment	10.48 (10.34, 10.60)^‡^	5.69 (5.51, 5.87)^‡^
Bowel incontinence	10.46 (10.37, 10.55)^‡^	4.53 (4.38, 4.68)^‡^
Cognitive status		
Intact/borderline	Reference	Reference
Mild/moderate impairment	3.89 (3.76, 4.01)^‡^	1.67 (1.55, 1.79)^‡^
Moderate- severe/severe impairment	11.73 (11.58, 11.87)^‡^	5.27 (5.10, 5.44)^‡^
Hearing impairment		
None	Reference	Reference
Hearing impaired	1.73 (1.55, 1.90)^‡^	0.03 (−0.08, 0.14)
Missing data	−0.56 (−1.80, 0.67)	0.66 (−0.15, 1.46)
Body mass index (BMI)		
BMI < 18.5	Reference	Reference
18.5 ≤ BMI ≤ 25	−2.02 (−2.24, −1.80)^‡^	−0.54 (−0.68, −0.40)^‡^
25 < BMI <30	−3.49 (−3.72, −3.26)^‡^	−0.87 (−1.03, −0.72)^‡^
BMI ≥ 30	−3.74 (−3.98, −3.50)^‡^	−0.59 (−0.75, −0.43)^‡^
Pain		
None	Reference	Reference
Less than daily pain	−0.70 (−0.85, −0.56)^‡^	0.29 (0.19, 0.39)^‡^
Daily or severe daily pain	−0.12 (−0.29, 0.04)	0.82 (0.70, 0.94)^‡^
Pressure ulcer	6.47 (6.23, 6.72)^‡^	2.67 (2.52, 2.82)^‡^
Urinary incontinence	10.50 (10.40, 10.61)^‡^	4.19 (4.04, 4.35)^‡^
Visual impairment		
None	Reference	Reference
Moderate impairment	3.09 (2.97, 3.22)^‡^	0.68 (0.59, 0.77)^‡^
Severe impairment	7.62 (7.40, 7.84)^‡^	2.49 (2.33, 2.65)^‡^
Prevalent Chronic Conditions		
Arthritis	−0.66 (−0.78, −0.54)^‡^	0.08 (−.0003, 0.15)
Asthma	−0.71 (−0.94, −0.48)^‡^	0.10 (−0.04, 0.24)
Cancer	−1.23 (−1.36, −1.11)^‡^	−0.12 (−0.19, −0.04)^†^
Chronic kidney disease	0.06 (0.08, 0.20)^‡^	0 .31 (0.22, 0.40)^‡^
Coronary artery disease	−0.86 (−0.98, −0.74)^‡^	−0.13 (−0.21, −0.05)^†^
Chronic obstructive pulmonary disease	−1.39 (−1.54, −1.25)^‡^	−0.07 (−0.17, 0.02)
Dementia	3.39 (3.22, 3.55)^‡^	−0.22 (−0.35, −0.10)^†^
Diabetes	−0.09 (−0.21, 0.04)	−0.06 (−0.14, 0.02)
Epilepsy	2.17 (1.94, 2.41)^‡^	0.47 (0.32, 0.61)^‡^
Heart failure	−0.24 (−0.38, −0.11)^‡^	0.36 (0.27, 0.46)^‡^
Limb paralysis or amputation	4.49 (4.29, 4.70)^‡^	1.78 (1.63, 1.93)^‡^
Mood disorder	0.53 (0.41, 0.65)^‡^	0.30 (0.22, 0.38)^‡^
Parkinson’s disease	2.87 (2.66, 3.07)^‡^	1.75 (1.63, 1.87)^‡^
Peripheral vascular disease	−0.14 (−0.34, 0.07)	0.03 (−0.10, 0.16)
Psychiatric conditions other than depression and dementia	−1.35 (−1.48, −1.22)^‡^	−0.42 (−0.50, −0.33)^‡^
Stroke	1.85 (1.73, 1.98)^‡^	0.46 (0.38, 0.55)^‡^
Random Effects		
*√ψ*	N/A	1.58 (1.50, 1.68)
*√θ*	N/A	4.90 (4.84, 4.96)
Derived Estimates		
*R* ^*2*^	N/A	0.627
*ρ*	N/A	0.095

Reference: Variable category is the reference group for all other categories within that variable

^†^ *p-*value <0.01

^‡^ *p-*value <0.0001

^§^Model 1: Adjusted for resident age, sex, marital status, pre-admission neighborhood income quintile, number of days since admission to long-term care home; includes random intercept for long-term care homes

*√* ***ψ***: Square root of between-long-term care home variance

*√* ***θ***: Square root of within-long-term care home variance

The null model of disability containing only random LTCH intercepts and no explanatory resident or LTCH variables had a within-LTCH variance of 66.91 and a between-LTCH variance of 4.16; variances from all multivariable models were compared to these values to estimate proportion of variance explained (*R* ^*2*^)

*R* ^*2*^: The proportional reduction in the estimated total residual variance compared to the null model (Model 1)

ρ: Proportion of variance that is explained by LTCH characteristics = ψ/(ψ + θ)

N/A: Not applicable because each coefficient in this column from distinct unadjusted bivariate regression with its own *√ψ, √θ, R* ^*2*^ and ρ

#### Geriatric syndromes and chronic conditions associated with disability

Balance impairment, urinary and bowel incontinence, pressure ulcer, severe visual impairment and severe cognitive impairment each had statistically significant independent associations with a minimum 2.5 point increase in disability (Table 3). Mild to moderate cognitive impairment, moderate visual impairment and daily or severe daily pain were also positively associated with more disability, but their effects were smaller, ranging from 0.59 to 1.79 (Table 3). Compared to being underweight, having a healthy body mass index (BMI) and being obese were both associated with lower disability; the protective effects of not being underweight were greatest in residents with overweight BMIs.

Compared to geriatric syndromes, chronic conditions had small associations with disability in multivariable models (Table 3). Exclusion of geriatric syndromes from the model resulted in increased effect size and statistical significance of chronic condition coefficients, but reduced the model *R* ^*2*^ from 62.7% to 11.2% (see Additional file 1: Table S6). Having Parkinson’s, heart failure, stroke, limb paralysis or amputation, kidney disease, or mood disorder were significantly associated with higher resident disability, however the size of these independent associations were smaller than those of geriatric syndromes, ranging from 0.22 to 1.93. Asthma, peripheral vascular disease and diabetes were not significantly associated with disability in multivariable models.

Dementia was strongly associated with higher disability in a bivariate model (Table 3), and in a model without geriatric syndromes (see Additional file 1: Table S6); this association between dementia and disability is reversed in Model 1, which also adjusted for cognitive status. Although bivariate analyses indicated a negative association between pain and disability, pain was positively associated with disability in fully adjusted analyses (Table 3); exploratory analyses revealed that the change in sign for pain occurred due to adjustment for coexisting geriatric syndromes and number of days since admission (data not shown). A similar reversal of a negative bivariate relationship between heart failure and disability occurred in multivariate models (Table 3), due to adjustment for number of days since admission (data not shown).

#### Effect modification by residents’ sex, age and cognitive status

As shown in Table 4, the estimated association between chronic conditions and geriatric syndromes with disability in the study sample did not differ in sub-samples of men, women, or individuals aged 74 to 94. The effect sizes of bowel incontinence, diabetes and cognitive status varied in the youngest (aged 65–74) and oldest (aged 95-plus) residents, however these differences were minor. Only 24% of residents in the sample did not suffer from moderate to severe cognitive impairment; in these people the association between pressure ulcer and limb paralysis or amputation and disability increased significantly. Conversely, co-existing dementia, visual impairment or bowel incontinence were associated more strongly with disability in those with cognitive impairment. Model estimates for all covariates included in sex-, age- and cognitive status-stratified versions of Models 1 can be found in Additional file 1: Table S7.

**Table 4:** Stratification by sex, age and cognitive status effects associations between geriatric syndromes, chronic conditions and disability

		Sex Stratified Models		Age Stratified Models				Cognitive Status-Stratified Models	
	Model 1	Females (*n =* 54,953)	Males (*n =* 22,212)	Age 65-74 (*n =* 7,859)	Age 75-84 (*n =* 25,703)	Age 85-94 (*n =* 36,676)	Age 95–105 (*n =* 6,927)	No cognitive impairment (*n =* 18,426)	Cognitive impairment present (*n =* 58,739)
Prevalent Geriatric Syndromes									
Balance impairment	5.69 (5.51, 5.87)^‡^	5.73 (5.52, 5.93)^‡^	5.51 (5.28, 5.74)^‡^	5.94 (5.57, 6.31)^‡^	5.71 (5.49, 5.93)^‡^	5.46 (5.25, 5.68)^‡^	5.42 (5.00, 5.85)^‡^	5.55 (5.31, 5.80)^‡^	5.95 (5.75, 6.16)^‡^
Bowel incontinence	4.53 (4.38, 4.68)^‡^	4.43 (4.26, 4.60)^‡^	4.77 (4.57, 4.97)^‡^	5.00 (4.66, 5.33)^‡^	4.61 (4.41, 4.82)^‡^	4.46 (4.28, 4.65)^‡^	3.98 (3.65, 4.32)^‡^	4.60 (4.35, 4.86)^‡^	5.43 (5.26, 5.60)^‡^
Cognitive status									
Intact/borderline	Reference	Reference	Reference	Reference	Reference	Reference	Reference	Reference	Reference
Mild/moderate impairment	1.67 (1.55, 1.79)^‡^	1.69 (1.55, 1.83)^‡^	1.67 (1.46, 1.88)^‡^	1.14 (0.85, 1.43)^‡^	1.51 (1.31, 1.70)^‡^	1.88 (1.71, 2.04)^‡^	1.86 (1.50, 2.22)^‡^	N/A	N/A
Moderate-severe/severe impairment	5.27 (5.10, 5.44)^‡^	5.40 (5.21, 5.59)^‡^	4.94 (4.67, 5.21)^‡^	4.21 (3.81, 4.61)^‡^	5.16 (4.91, 5.41)^‡^	5.57 (5.34, 5.79)^‡^	5.35 (4.88, 5.81)^‡^	N/A	N/A
Hearing impairment									
None	Reference	Reference	Reference	Reference	Reference	Reference	Reference	Reference	Reference
Hearing impaired	0.03 (−0.08, 0.14)	0.02 (−0.12 0.15)	0.07 (−0.12, 0.26)	−0.13 (−0.71, 0.45)	−0.04 (−0.26, 0.18)	−0.04 (−0.18, 0.11)	0.26 (0.002, 0.51)^*^	0.43 (0.14, 0.72)^†^	0.08 (−0.04, 0.20)
Missing data	0.66 (−0.15, 1.46)	0.33 (−0.67, 1.34)	1.21 (−0.09, 2.52)	1.85 (−0.79, 4.49)	0.005 (−1.21, 1.22)	1.18 (−0.21, 2.57)	0.007 (−3.06, 3.08)	0.32 (−1.27, 1.90)	0.74 (−0.16, 1.64)
Body mass index (BMI)									
BMI < 18.5	Reference	Reference	Reference	Reference	Reference	Reference	Reference	Reference	Reference
18.5 ≤ BMI ≤ 25	−0.54 (−0.68, −0.40)^‡^	−0.56 (−0.71, −0.41)^‡^	−0.52 (−0.84, −0.21)^†^	−0.03 (−0.57, 0.52)	−0.46 (−0.72, −0.19)^†^	−0.67 (−0.86, −0.49)^‡^	−0.55 (−0.89, −0.20)^†^	−0.80 (−1.16, −0.43)^‡^	−0.51 (−0.66, −0.36)^‡^
25 < BMI <30	−0.87 (−1.03, −0.72)^‡^	−0.83 (−0.99, −0.66)^‡^	−1.04 (−1.36, −0.71)^‡^	−0.69 (−1.24, −0.14)^*^	−0.82 (−1.09, −0.54)^‡^	−0.96 (−1.17, −0.76)^‡^	−0.83 (−1.23, −0.44)^‡^	−1.04 (−1.42, −0.67)^‡^	−0.97 (−1.14, −0.80)^‡^
BMI ≥ 30	−0.59 (−0.75, −0.43)^‡^	−0.52 (−0.69, −0.34)^‡^	−0.89 (−1.24, −0.53)^‡^	−0.68 (−1.23, −0.12)^*^	−0.55 (−0.84, −0.26)^‡^	−0.62 (−0.84, −0.41)^‡^	−0.31 (−0.80, 0.18)	−0.50 (−0.89,-0.10)^†^	−0.98 (−1.16, −0.81)^‡^
Pain									
None	Reference	Reference	Reference	Reference	Reference	Reference	Reference	Reference	Reference
Less than daily pain	0.29 (0.19, 0.39)^‡^	0.25 (0.13, 0.36)^‡^	0.39 (0.22, 0.57)^‡^	0.26 (−0.03, 0.54)	0.18 (0.03, 0.34)^*^	0.34 (0.20, 0.48)^‡^	0.19 (−0.09, 0.48)	0.50 (0.30, 0.69)^‡^	−0.007 (−0.12, 0.11)
Daily or severe daily pain	0.82 (0.70, 0.94)^‡^	0.78 (0.64, 0.92)^‡^	0.90 (0.68, 1.13)^‡^	0.70 (0.35, 1.05)^‡^	0.76 (0.57, 0.95)^‡^	0.86 (0.69, 1.02)^‡^	0.67 (0.28, 1.05)^†^	0.81 (0.59, 1.04)^‡^	0.62 (0.47, 0.76)^‡^
Pressure ulcer	2.67 (2.52, 2.82)^‡^	2.70 (2.52, 2.87)^‡^	2.59 (2.32, 2.86)^‡^	3.03 (2.57, 3.48)^‡^	2.70 (2.45, 2.95)^‡^	2.63 (2.42, 2.84)^‡^	2.39 (1.98, 2.81)^‡^	3.34 (2.98, 3.71)^‡^	2.78 (2.62, 2.94)^‡^
Urinary incontinence	4.19 (4.04, 4.35)^‡^	4.30 (4.12, 4.49)^‡^	3.97 (3.76, 4.19)^‡^	4.00 (3.66, 4.34)^‡^	4.19 (3.98, 4.40)^‡^	4.28 (4.08, 4.49)^‡^	4.06 (3.66, 4.45)^‡^	4.48 (4.25, 4.71)^‡^	4.28 (4.10, 4.46)^‡^
Visual impairment									
None	Reference	Reference	Reference	Reference	Reference	Reference	Reference	Reference	Reference
Moderate impairment	0.68 (0.59, 0.77)^‡^	0.68 (0.57, 0.78)^‡^	0.70 (0.55, 0.85)^‡^	0.73 (0.48, 0.98)^‡^	0.64 (0.50, 0.79)^‡^	0.67 (0.55, 0.79)^‡^	0.80 (0.53, 1.07)^‡^	0.53 (0.33, 0.73)^‡^	0.99 (0.88, 1.09)^‡^
Severe impairment	2.49 (2.33, 2.65)^‡^	2.45 (2.27, 2.63)^‡^	2.58 (2.30, 2.86)^‡^	2.82 (2.38, 3.26)^‡^	2.72 (2.45, 3.00)^‡^	2.39 (2.18, 2.60)^‡^	2.21 (1.83, 2.59)^‡^	1.98 (1.49, 2.48)^‡^	3.50 (3.32, 3.67)^‡^
Prevalent Chronic Conditions									
Arthritis	0.08 (−.0003, 0.15)	0.09 (−0.007, 0.18)	0.04 (−0.09, 0.16)	−0.16 (−0.40, 0.08)	0.10 (−0.03, 0.23)	0.12 (0.006, 0.23)^*^	0.13 (−0.11, 0.38)	0.13 (−0.05, 0.32)	−0.10 (−0.18,−0.008)^*^
Asthma	0.10 (−0.04, 0.24)	0.09 (−0.06, 0.24)	0.17 (−0.11, 0.45)	0.03 (−0.42, 0.48)	0.10 (−0.14, 0.34)	0.20 (−0.001, 0.40)	−0.11 (−0.62, 0.39)	0.07 (−0.22, 0.35)	0.08 (−0.07, 0.24)
Cancer	−0.12 (−0.19, −0.04)^†^	−0.15 (−0.25, −0.06)^†^	−0.06 (−0.20, 0.06)	−0.09 (−0.36, 0.18)	−0.16 (−0.29, −0.03)^*^	−0.08 (−0.20, 0.02)	−0.17 (−0.43, 0.08)	−0.20 (−0.38, −0.03)^*^	−0.21 (−0.30, −0.12)^‡^
Chronic kidney disease	0.31 (0.22, 0.40)^‡^	0.26 (0.15, 0.37)^‡^	0.40 (0.24, 0.55)^‡^	0.39 (0.09, 0.68)^*^	0.32 (0.17, 0.48)^‡^	0.31 (0.18, 0.44)^‡^	0.32 (0.02, 0.62)^*^	0.22 (0.03, 0.41)^*^	0.26, (0.15, 0.36)^‡^
Coronary artery disease	−0.13 (−0.21, −0.05)^†^	−0.11 (−0.20, −0.02)^*^	−0.18 (−0.32, −0.04)^*^	−0.29 (−0.56, −0.02)^*^	−0.04 (−0.17, 0.10)	−0.15 (−0.26, −0.04)^*^	−0.19 (−0.44, 0.05)	−0.25 (−0.43, −0.08)^†^	−0.17 (−0.26, −0.07)^‡^
Chronic obstructive pulmonary disease	−0.07 (−0.17, 0.02)	−0.09 (−0.21, 0.02)	−0.04 (−0.20, 0.12)	−0.09 (−0.38, 0.21)	−0.30 (−0.47, −0.14)^‡^	0.08 (−0.05, 0.21)	0.11 (−0.21, 0.44)	−0.16 (−0.35, 0.03)	−0.23 (−0.34, −0.12)^‡^
Dementia	−0.22 (−0.35, −0.10)^†^	−0.32 (−0.47, −0.17)^‡^	0.02 (−0.20, 0.23)	−0.41 (−0.72, −0.10)^*^	−0.39 (−0.61, −0.17)^‡^	−0.10 (−0.29, 0.08)	0.21 (−0.16, 0.59)	−0.25 (−0.43, −0.06)^†^	0.23 (0.06, 0.40)^†^
Diabetes	−0.06 (−0.14, 0.02)	−0.03 (−0.13, 0.07)	−0.12 (−0.25, 0.01)	−0.24 (−0.49, 0.009)	−0.10 (−0.23, 0.03)	−0.004 (−0.12, 0.12)	0.32 (0.04, 0.60)^*^	−0.34 (−0.52, −0.16)^‡^	−0.12 (−0.21, −0.03)^*^
Epilepsy	0.47 (0.32, 0.61)^‡^	0.61 (0.44, 0.78)^‡^	0.20 (−0.03, 0.44)	0.47 (0.16, 0.77)^†^	0.36 (0.14, 0.58)^†^	0.60 (0.37, 0.84)^‡^	−0.05 (−0.77, 0.67)	0.13 (−0.23, 0.49)	0.62 (0.46, 0.79)^‡^
Heart failure	0.36 (0.27, 0.46)^‡^	0.36 (0.25, 0.47)^‡^	0.37 (0.20, 0.53)^‡^	0.31 (−0.009, 0.63)	0.41 (0.24, 0.58)^‡^	0.35 (0.22, 0.48)^‡^	0.34 (0.06, 0.61)^*^	0.47 (0.29, 0.65)^‡^	0.20 (0.09, 0.31)^‡^
Limb paralysis or amputation	1.78 (1.63, 1.93)^‡^	1.81 (1.63, 2.00)^‡^	1.79 (1.55, 2.02)^‡^	1.59 (1.27, 1.91)^‡^	1.93 (1.70, 2.17)^‡^	1.77 (1.55, 1.99)^‡^	1.57 (1.11, 2.04)^‡^	2.54 (2.24, 2.83)^‡^	1.44 (1.27, 1.60)^‡^
Mood disorder	0.30 (0.22, 0.38)^‡^	0.32 (0.23, 0.42)^‡^	0.26 (0.12, 0.41)^‡^	−0.10 (−0.33, 0.14)	0.20 (0.08, 0.33)^†^	0.44 (0.33, 0.55)^‡^	0.38 (0.12, 0.64)^†^	0.42 (0.23, 0.60)^‡^	0.15 (0.06, 0.24)^†^
Parkinson’s disease	1.75 (1.63, 1.87)^‡^	1.79 (1.63, 1.95)^‡^	1.72 (1.53, 1.91)^‡^	1.61 (1.25, 1.97)^‡^	1.87 (1.69, 2.06)^‡^	1.66 (1.46, 1.86)^‡^	1.66 (1.09, 2.23)^‡^	2.18 (1.89, 2.47)^‡^	1.54 (1.41, 1.67)^‡^
Peripheral vascular disease	0.03 (−0.10, 0.16)	0.11 (−0.05, 0.27)	−0.10 (−0.31, 0.11)	−0.24 (−0.65, 0.17)	0.01 (−0.21, 0.23)	0.13 (−0.06, 0.31)	0.001 (−0.44, 0.45)	−0.18 (−0.43, 0.06)	−0.03 (−0.18, 0.13)
Psychiatric conditions other than depression and dementia	−0.42 (−0.50, −0.33)^‡^	−0.39 (−0.49, −0.29)^‡^	−0.46 (−0.62, −0.31)^‡^	−0.61 (−0.84, −0.39)^‡^	−0.38 (−0.52, −0.24)^‡^	−0.35 (−0.48, −0.23)^‡^	−0.20 (−0.49, 0.09)	−0.65 (−0.83, −0.46)^‡^	−0.39 (−0.48, −0.30)^‡^
Stroke	0.46 (0.38, 0.55)^‡^	0.48 (0.37, 0.58)^‡^	0.45 (0.29, 0.60)^‡^	0.39 (0.13, 0.66)^†^	0.48 (0.34, 0.63)^‡^	0.45 (0.32, 0.58)^‡^	0.56 (0.28, 0.83)^‡^	0.68 (0.49, 0.86)^‡^	0.35 (0.25, 0.44)^‡^

Model 1: Adjusted for resident age, sex, marital status, pre-admission neighborhood income quintile, number of days since admission to long-term care home; includes random intercept for long-term care homes

*N/A* Not applicable; variable not included in indicated model

Reference: Variable category is the reference group for all other categories within that variable

^*^ *p-*value <0.05

^†^ *p-*value <0.01

^‡^ *p-*value <0.0001

#### Long-term care home characteristics associated with resident disability

Residents’ demographic characteristics and morbidity explained 62.7% of the variance in disability score. Although a likelihood ratio test indicated that there were statistically significant between-LTCH differences in resident disability (χ^2^ = 3389.1, *p <* 0.000), LTCH variables such as intensity of rehabilitation services or ownership type, explained only an additional 2% of the variance.

### Sensitivity analyses

Sensitivity analyses that removed geriatric syndromes and chronic conditions from multivariable models (see Additional file 1: Table S6) show that geriatric syndromes explained a large amount of the variance in residents’ disability, including some of the effects of chronic conditions. In fact, a sensitivity analysis in which only four geriatric syndromes (balance impairment, urinary and bowel incontinence and cognitive impairment) were modelled explained 59.9% of the variance in disability (see Additional file 1: Table S6). Use of fixed effects to adjust for clustering within LTCHs did not significantly change any model estimates (see Additional file 1: Table S8); proportion of variance in residents’ disability attributable to between-home differences was 10.3% in a fixed effects Model 1 versus 9.5% in the random effects Model 1. A version of Model 1 without random or fixed effects for LTCHs explained just as much variance in disability as models that accounted for differences between LTCHS (see Additional file 1: Table S8), further verifying the absence of LTCH effects on resident disability. Use of only administrative claims data to code chronic conditions reduced the effects of limb paralysis or amputation and mood disorders, rendering them non-significant, while significantly increasing the effect of stroke (see Additional file 1, Table S9). Use of only RAI-MDS chronic condition codes did not significantly change the effects of any chronic conditions or geriatric syndromes (see Additional file 1: Table S9). Exclusion of the 9,302 residents (12% of sample) whose data were from admission assessments had no effect on findings from Model 2 (see Additional file 1: Table S9).

## Discussion

### Geriatric syndromes explain major differences in disability

Geriatric syndromes were much more strongly associated with disability in LTCH residents than were chronic conditions; their removal from Model 1 reduced the *R* ^*2*^ from 62.7% to 11.2% (see Additional file 1: Table S6), showing that they explain approximately 50% of unique variation in resident disability in this population-based sample. The geriatric syndromes that were most strongly associated with disability were balance impairment, cognitive impairment and urinary and bowel incontinence. Characteristics of LTCHs accounted for less than 2% of the variance in resident disability once resident characteristics were considered. These findings suggest that residents and their geriatric syndromes—not the LTCHs in which they live—may be appropriate targets of interventions to reduce disability, and that such interventions may need to differ for cognitively impaired versus unimpaired residents.

### Mechanisms for geriatric syndrome, chronic condition and LTCH effects

The Disablement Process Model [20] that guided hypothesis generation and analysis for this study is also instructive in understanding its main findings. The strong association between geriatric syndromes and disability was insensitive to adjustment for coexisting chronic conditions, whereas effects of chronic conditions diminished or were rendered non-significant after adjustment for coexisting geriatric syndromes. A possible mechanism for this finding is that geriatric syndromes mediate some of the effects of chronic conditions on disability. For example, limb paralysis or amputation is strongly associated with disability, but some of this association may be mediated by daily pain. This possible mechanism should be further explored in mediation analyses. An alternative explanation is that geriatric syndromes are proxy measures for disease severity or close proximity to end of life, both of which are associated with disability but not directly measured in our study.

Although there was significant variation in the distribution of LTCH characteristics, descriptive analyses showed that these variations were not associated with corresponding variations in resident disability. This is the likely cause of the lack of explanatory power LTCH characteristics had in models of resident disability.

### Understanding effect modification by age, sex and cognitive status

The consistency of chronic condition and geriatric syndrome effects on disability across age and sex strata may represent a nullification of age- and sex- effect modification due to the advanced age and morbidity of LTCH residents. However, it is also possible that females and the oldest old residents in whom chronic conditions and geriatric syndromes were the most strongly associated with disability were under-represented in this cross-sectional sample due to early mortality. We found that some chronic conditions and geriatric syndromes effected residents who were cognitively impaired differently than those who were cognitively intact. Cognitive impairment may exacerbate the effect of prevalent conditions and syndromes due to its impact on older adults’ ability to self-care [39], whereas activity-limiting conditions like limb paralysis and Parkinson’s may have stronger effects among cognitively intact residents due to their lower overall disability.

### Findings in the context of existing evidence

The dominance of geriatric syndromes over chronic conditions as determinants of health status has recently been demonstrated in community-dwelling older adults [40] but our exploration of this relationship in LTCH residents offers new insight. Other studies of geriatric syndromes’ effect on disability in LTCH residents adjusted for the number of chronic conditions that patients had, rather than examining the effects of specific chronic conditions alongside specific geriatric syndromes [17, 18, 33, 41]. Our inclusion of specific chronic conditions in multivariable models revealed that effects of some chronic conditions (e.g. dementia) were particularly sensitive to adjustment for coexisting geriatric syndromes in models of disability.

While we found a stronger effect of pressure ulcer on disability in cognitively intact residents, the effect of bowel incontinence and visual impairment on disability was significantly stronger in cognitively impaired residents in our sample. These mixed results align with existing evidence, some of which supports exacerbated effects of chronic conditions among cognitively impaired older adults [15] and some of which shows worse effects among cognitively intact older adults [32]. Compared to being underweight (BMI <18.5), having an overweight BMI (BMI 25–30) was associated with the lowest level of disability in multivariable models. These findings mirror findings regarding the association of BMI with mortality: results of a recent meta-analysis of more than 30.3 million people in 230 cohort studies found that the lowest mortality was observed among those with a BMI of approximately 25 [42]. Similar to the association between underweight BMI and increased mortality [42], the relationship between BMI <18.5 and disability in this study may be confounded by low body weight resulting from chronic disease [43]. Our adjustment for 16 chronic conditions and nine geriatric syndromes addresses much of this potential confounding, however longitudinal studies of this association are needed.

The proportion of variance in resident disability (<2%) explained by LTCH characteristics in this sample is smaller than the 8–25% variance in ADL-LFS found by Phillips et al. in their studies of 1,334 American LTCHs [17, 41]. We hypothesize that this difference occurred because we adjusted for significantly more chronic conditions and geriatric syndromes than Phillips et al., and explained a larger proportion of total model variance (*R* ^*2*^ = 64.2%) than Phillips et al. achieved (*R* ^*2*^ = 18%) [41], thus reducing variance attributed LTCHs. The weak effects of specific LTCH variables in our study is consistent with another study of LTCH effects on disablement in LTCH residents [33], as well as equivocal evidence for the relationship between LTCH characteristics and other resident health outcomes [12, 44].

### Strengths

This study used health administrative data in a single-payer health care system to study the relationships between resident morbidity, LTCH characteristics and disability in a large, representative sample with adjustment for multiple confounders. Our large sample size also allowed for examination of effects among strata of putative effect modifiers that were larger than many studies’ main samples. In contrast with most studies in LTCH residents that use either validated administrative claims algorithms or RAI-MDS active diagnoses to identify chronic conditions, we combined these measures and tested the sensitivity of our findings to this choice. Although claims data tend to be more sensitive for the detection of some diagnoses (e.g. heart failure, arthritis), RAI-MDS assessments are more sensitive to other conditions (e.g. Alzheimer’s, hip fracture) [45]. Our findings suggest that using combined chronic condition measures from both data sources yields findings that are fairly comparable to those generated using only one. Existing studies that examine the relationship between morbidity and disability either do not include specific chronic conditions in models [17, 18, 33, 41], or do not examine the sensitivity of model estimates to adjustment for geriatric syndromes [16]. By doing both, we produced robust empirical findings while also testing a theoretical extension of a heavily used conceptual framework. The absence of multicollinearity in our large multivariable models supports future use of the Disablement Process Model [20] as a guide to identify conceptually distinct variables in disability research.

### Limitations

Due to the cross-sectional nature of our study, we cannot make causal inferences regarding the associations that we report. Our sample also captures residents at different stages of their LTCH journey; because the magnitude of association between specific chronic conditions and geriatric syndromes and disability may change over time since admission, we adjusted multivariable models for the duration of time residents had been in their LTCH. There were several geriatric syndromes that were not included in this study for conceptual and methodological reasons. Extant conceptualizations and administrative data measures of frailty include both the outcome (disability) and the exposures (symptoms, signs, disease) of interest in this study [46, 47], therefore frailty was not included in multivariable models despite being a well-known geriatric syndrome. Sarcopenia—defined as low muscle mass and either reduced strength or physical performance [48] – was not measured in this study, however we measured BMI, which is an excellent proxy for sarcopenia in age-adjusted models among older adults [49]. Polypharmacy is another geriatric syndrome that was excluded from this analysis because it is a marker of underlying health status that exists along the causal pathway between the exposures of interest (chronic conditions and geriatric syndromes) and health outcomes (disability) in this study [50]. Delirium and falls are important geriatric syndromes that were excluded from this cross-sectional study due to their acute nature and the longitudinal data required to examine their effects; however, our inclusion of geriatric syndromes associated with delirium and falls (i.e. cognitive status and balance impairment) likely accounted for much of the variance in disability associated with these acute health events.

We did not have data on numerous LTCH characteristics potentially associated with residents’ disability (e.g. staffing levels [10] and immunization rates [51]), therefore interpretation of the effects for the few LTCH variables we did measure (e.g. for-profit ownership) should be tempered by the knowledge that these variables may be absorbing variance from unmeasured variables. However, we did replicate our findings in models with fixed effects for LTCHs and thus verified that they were not due our inability to measure relevant LTCH characteristics in our random effects models.

## Conclusions

Our findings show that geriatric syndromes explain more variation in resident disability than chronic conditions and features of LTCHs combined. These findings suggest that residents and their geriatric syndromes—not the LTCHs in which they live—may be the appropriate target of interventions to reduce disability, and that such interventions may need to differ for cognitively impaired versus unimpaired residents. These associations should be further explored in longitudinal studies.

## Additional file

Additional file 1: Table S1. (Disablement Process Model definitions and examples); **Table S2.** (Items and Possible Responses in the RAI-MDS ADL Long Form Scale); **Table S3.** (Chronic Conditions and Diagnostic Criteria Used to Identify them in Claims and Health Assessment Databases); **Table S4.** (Geriatric Syndromes and Diagnostic Criteria Used to Identify them in the CCRS Database); **Table S5.** (All Variable Coefficient Estimates from Models 1 and 2); **Table S6.** (Model 1 Excluding Chronic Conditions, Geriatric Syndromes); **Table S7.** (All Variable Coefficient Estimates from Stratified Versions of Model 1); **Table S8.** (Sensitivity of Model 1 Findings to Unmeasured LTCH Variables and Lack of adjustment for Long-Term Care Homes); **Table S9.** (Sensitivity of Model 1 Findings to Coding of Chronic Conditions); **Table S10.** (Sensitivity of Model 2 Findings to Exclusion of Admission Assessments). (DOCX 87 kb)

## Abbreviations

ADL LFS: Activities of daily living long-form score
ADLs: Activities of daily living
BMI: Body Mass Index
CCRS: Continuing care reporting system
DAD: Discharge abstract database
ICES: Institute for clinical evaluative sciences
LTCH: Long-term care home
MOHLTC: Ministry of health and long-term care
OHIP: Ontario health insurance plan
RAI-MDS: Resident assessment instrument minimum dataset 2.0
RPDB: Registered persons database
VIF: Variance inflation factor
