# Supplementary material for: Disability in long-term care residents explained by prevalent geriatric syndromes, not long-term care home characteristics: a cross-sectional study
Source: BMC Geriatr. 2017 Feb 10;17:49. doi: 10.1186/s12877-017-0444-1 (PMC5301427; doi:10.1186/s12877-017-0444-1)
Supplement: Additional file 1: Table S1. — (Disablement Process Model definitions and examples); Table S2. (Items and Possible Responses in the RAI-MDS ADL Long Form Scale); Table S3. (Chronic Conditions and Diagnostic Criteria Used to Identify them in Claims and Health Assessment Databases); Table S4. (Geriatric Syndromes and Diagnostic Criteria Used to Identify them in the CCRS Database); Table S5. (All Variable Coefficient Estimates from Models 1 and 2); Table S6. (Model 1 Excluding Chronic Conditions, Geriatric Syndromes); Table S7. (All Variable Coefficient Estimates from Stratified Versions of Model 1); Table S8. (Sensitivity of Model 1 Findings to Unmeasured LTCH Variables and Lack of adjustment for Long-Term Care Homes); Table S9. (Sensitivity of Model 1 Findings to Coding of Chronic Conditions); Table S10. (Sensitivity of Model 2 Findings to Exclusion of Admission Assessments). (DOCX 87 kb) [file 12877_2017_444_MOESM1_ESM.docx]

## Table S1

*Disablement Process Model definitions and examples [*[*1*](#_ENREF_1)*]*

| **Term** | **Definition** | **Examples** |
| --- | --- | --- |
| **Pathology** | Biochemical and physiological abnormalities that are detected and medically labeled as disease or injury. | |
|  | **Sub-clinical pathology^*^**: detectable biochemical and physiological abnormalities not associated with impairment. | - hypocholesteremia  - reduced cardiac ejection fraction |
|  | **Acute pathology**^*^: Short-term diseases and injuries, usually lasting three months or less. | - fall  - delirium  - lower respiratory tract infection |
|  | **Chronic pathology**: Progressive diseases, injuries with chronic sequelae, and enduring structural/sensory abnormalities | - arthritis  - chronic obstructive pulmonary disease  - heart failure |
| **Impairment** | Dysfunction and significant structural abnormalities in specific body systems that have consequences for physical, mental or social function. | - cognitive impairment  - chronic pain  - visual impairment |
| **Functional limitation** | Restrictions in performing physical and mental actions used in daily life by one’s age-sex group.  Refers to individual capability without reference to situational requirements. | Difficulty performing any of following actions:  - walking specified distances  - lifting objects of specified weight  - climbing stairs |
| **Disability** | Difficulty with or dependence on others to conduct activities of daily living (ADLs), measured at a single point in time. | Difficulty with or dependence on others to conduct ADLs such as:  - bathing  - dressing  - transferring from bed to chair  - toileting  - grooming  - feeding |
| **Disablement** | Intensifying disability over at least two time points. | Requiring additional assistance to conduct – or becoming newly dependent in – one of the self-care ADLs above. |
| **Extra-Individual factors** | Factors that operate outside or external to a person and affect the Disablement Process. Can be grouped into one of the following categories: | |
|  | Medical care and rehabilitation | - surgery  - physical therapy |
|  | Medications and other therapeutic regimens | - drugs taken  - recreational therapy |
|  | External supports | - receipt of personal assistance e.g. meals on wheels  - use of special equipment and devices |
|  | Built physical and social environment | - structural modification at home  - health insurance and access to medical care  - laws and regulations |
| **Intra-Individual Factors** | Factors that operate within a person and affect the Disablement Process. Can be grouped into one of the following categories: | |
|  | - Demographic characteristics  - Biological attributes  - Lifestyle and behavioral factors  - Psychosocial attributes | - age, sex, race - genetics  - smoking, physical activity  - beliefs, religiosity, socioeconomic status |

^*^Sub-clinical and acute pathologies were not examined in this study due to lack of available data on sub-clinical pathologies and inability to study acute (incident) exposures in cross-sectional data.

## Table S2

*Items and Possible Responses in the RAI-MDS ADL Long Form Scale*

| **Item** | **Description** |
| --- | --- |
| **Bed mobility** | Includes how a resident moves and turns their body position while in bed. |
| **Transfer** | Includes how a resident moves between surfaces such as bed and chair. |
| **Locomotion** | Includes how a resident moves between locations in their room and the corridor outside their room. |
| **Dressing** | Includes how a resident puts on, fastens and takes off all items of street clothing. |
| **Eating** | Relates to how a resident eats and drinks, including other means of nourishment intake, such as tube feeding. |
| **Toilet use** | This includes how a resident uses a toilet, commode, bedpan or urinal and transfer on and off a toilet. |
| **Personal hygiene** | Relates to how personal hygiene is maintained, including combing hair, brushing teeth, washing and drying face and hands. Excludes baths and showers. |
| Categories of response to each item include:  **0**: total independence or no or little help with activity.  **1**: supervision provided 3 or more times during last 7 days.  **2**: limited assistance by staff with the resident highly involved in the activity. **3**: extensive assistance by staff with the resident performing part of the activity.  **4**: total dependence/full staff participation in activity during the entire 7 days OR activity did not occur during past 7 days. | |

## Table S3

*Chronic Conditions and Diagnostic Criteria Used to Identify them in Claims and Health Assessment Databases*

| **Chronic conditions** | **ICD-9** *OHIP or CIHI-DAD* | **ICD-10** *CIHI-DAD* | **RAI-MDS**  *CCRS_LTC* |
| --- | --- | --- | --- |
| **Arthritis: osteo, rheumatoid and others** | 274, 710, 711, 714-16, 718, 720, 727-729, 739 | M00-M03, M05-M07,M10-M25, M30-M36, M65-M73, M75-M77, M79 | I1L: arthritis |
| **Asthma** | 493 | J45 | I1JJ: asthma |
| **Cancer** | 140-165, 170-176, 179-208,  210-239 | C00-C26, C30-C34, C37-C41, C43-C58, C60-86, C88, C91-97 | I1RR: cancer |
| **Coronary artery disease,** (including myocardial infarction) | 410-414 | I20-I25 | I1D: arteriosclerotic heart disease |
| **Chronic obstructive pulmonary disease** | 491, 492, 496 | J41-J44 | I1KK: emphysema/COPD |
| **Dementia**  (including Alzheimer’s) | **OHIP**: 290, 331, 797  **DAD**: 290, 294.1, 294.8, 294.9, 331.0, 331.1, 331.2, 797 | F000, F001, F002, F009-F013, F018-F024, F028, F03, F051, F065, F066, F068, F069, F09, G300 -G301, G308-G311, R54 | I1R: Alzheimer’s disease  I1V: Dementia other than Alzheimer’s disease |
| **Diabetes (Type 1 and 2)** | 250 | E10-E14 | I1A: Diabetes mellitus |
| **Epilepsy** | 345 | G40-G41 | I1CC: Seizure disorder |
| **Heart failure** | 428 | I500, I501, I509 | I1F: Congestive heart failure |
| **Limb paralysis or amputation** | 896, 897 | G82, G83, S48, S58, S68.3, S68.4, S68.8, S68.9, S78, S88, T05, T116, T136 | I1N: Missing limb (e.g. amputation)  I1W: hemiplegia/hemiparesis  I1Z: Paraplegia  I1BB: Quadriplegia |
| **Mood disorders** | **OHIP:** 296 311  **DAD:** 296.2, 296.3, 296.5. 311 | F30-F34 F38 F39 | I1GG: depression  I1HH: manic depressive (bipolar) |
| **Parkinson’s disease** | 332 | G20-G22 | I1AA: Parkinson’s disease |
| **Peripheral vascular disease** | **OHIP**: 440 **DAD**: 440.2 | I70.2 | I1J: Peripheral vascular disease |
| **Psychiatric conditions other than depression and dementia** | 291-292, 295, 297-298, 300-301, 303-310, 312, 315-319 | F04, F06, F07, F10-F25, F28-F29, F40-F45, 48  50-55, F59-F63, F68-F73,  F78-F84, F88-F91,  F94-F95, F98-F99  **EXCEPT:** F063, F065, F066, F068, F069 | I1FF: anxiety disorder  I1II: schizophrenia |
| **Renal disease** | 583-586, 592, 593 | N00-N08, N11, N13-14, N16-N23 | I1UU: renal failure |
| **Stroke** | 430-432, 434, 436 | I60-I64 | I1U: Cerebrovascular accident (stroke) |

## Table S4

*Geriatric Syndromes and Diagnostic Criteria Used to Identify them in the CCRS Database*

| **Geriatric Syndromes** | **CCRS_LTC Variable** | **Re-Coding of Variable for Study** |
| --- | --- | --- |
| **Balance impairment** | **G3A** | **Based on a test for balance while standing.  0**: **Not balanced impaired** - G3A = 0 or 1  G3A =0: Maintained position as required   during test  G3A =1: Unsteady, but able to rebalance self  without physical support  **1**: **Balance Impaired** - G3A = 2 or 3  G3A =2: Partial physical support during test;  or stands but does not follow directions for   test  G3A = 3: Not able to attempt test without  physical help |
| **BMI** | **K2A:** Resident’s height in centimeters.  **K2B**: Resident’s weight in kilograms (measured within 30 days of assessment). | Derived variable:  BMI = bodyweight in kg/(height in meters)^2^  = **K2B/(K28/100)^2^** |
| **Bowel incontinence** | **H1A** | **0**: Continent - H1A = 0 or 1 **1**: Bowel incontinent - H1A = 2, 3, 4 |
| **Cognitive status** | **CPS_CC** | **0**: Intact/ Borderline Intact - CPS_CC = 0 or 1  **1**: Mild or mod. Impaired - CPS_CC = 2 or 3 **2**: Mod. severely Impaired - CPS_CC = 4  **3**: Severely Impaired - CPS_CC = 5 or 6 |
| **Hearing impairment** | **C1** | **0**: Not highly impaired - C1 = 0, 1  **1**: Impaired C1 = 2, 3 |
| **Pain** | **PAIN_CC** | **0**: No pain  **1**: Less than daily pain  **2**: Daily pain but not severe  **3**: Severe daily pain |
| **Pressure ulcer** | **M2A** | **0:** No pressure ulcer - M2A = 0 or 1  **1:** Pressure ulcer present - M2A = 2, 3 or 4 |
| **Urinary incontinence** | **H1B** | **0**: Continent - H1B = 0, 1 **1**: Urine incontinent - H1B = 2, 3, 4 |
| **Visual impairment** | **D1** | **0** – Adequate vision - D1 = 0  **1** – Impaired to mod. Impaired - D1 = 1, 2  **2** – Highly or severely *impaired* - D1 = 3, 4 |

## Table S5

*All Variable Coefficient Estimates from Models 1 and 2*

|  | | **Unadjusted Bivariate Regressions** | | **Model 1**^§^ | | **Model 2**^\|\|^ |
| --- | --- | --- | --- | --- | --- | --- |
|  | | Estimate (95% CI) | | Estimate (95% CI) | | Estimate (95% CI) |
| Constant | |  | | 4.07 (3.71, 4.44)^‡^ | | 2.70 (2.18, 3.22)^‡^ |
| **Resident Characteristics** |  | |  | |  | |
| Age |  | |  | |  | |
| 65 – 74 | | Reference | | Reference | | Reference |
| 75 – 84 | | 0.76 (0.55, 0.98)^‡^ | | 0.04 (-0.08, 0.17) | | 0.04 (-0.08, 0.16) |
| 85 – 94 | | 1.02 (0.81, 1.22)^‡^ | | 0.18 (0.04, 0.31)^*^ | | 0.18 (0.04, 0.31)^*^ |
| 95+ | | 2.43 (2.16, 2.70)^‡^ | | 0.61 (0.42, 0.80)^‡^ | | 0.61 (0.42, 0.79)^‡^ |
| Sex |  | |  | |  | |
| Female | | Reference | | Reference | | Reference |
| Male | | -1.03 (-1.16, -0.90)^‡^ | | -0.37 (-0.46, -0.28) ^‡^ | | -0.36 (-0.46, -0.27)^‡^ |
| Marital Status |  | |  | |  | |
| Married | | Reference | | Reference | | Reference |
| Widowed | | -0.92 (-1.06, -0.78)^‡^ | | -0.41 (-0.50, -0.31)^‡^ | | -0.41 (-0.50, -0.31)^‡^ |
| Never married,   separated or   divorced | | -2.21 (-2.40, -2.01)^‡^ | | -0.60 (-0.73, -0.48)^‡^ | | -0.60 (-0.73, -0.48)^‡^ |
| Missing data on   marital status | | -1.23 (-1.73, -0.73)^‡^ | | -0.64 (-0.96, -0.32)^‡^ | | -0.64 (-0.97, -0.32)^‡^ |
| Pre-LTCH Neighborhood Income Quintile |  | |  | |  | |
| 1 (low) | | Reference | | Reference | | Reference |
| 2 | | 0.56 (0.37, 0.75)^‡^ | | 0.13 (0.01, 0.26)^*^ | | 0.12 (0.002, 0.25)^*^ |
| 3 | | 0.52 (0.33, 0.71)^‡^ | | 0.19 (0.07, 0.31)^†^ | | 0.18 (0.06, 0.30)^†^ |
| 4 | | 0.96 (0.76, 1.15)^‡^ | | 0.29 (0.17, 0.41)^‡^ | | 0.27 (0.15, 0.39)^‡^ |
| 5 (high) | | 0.95 (0.74, 1.15)^‡^ | | 0.23 (0.10, 0.37)^†^ | | 0.23 (0.09, 0.36)^†^ |
| Missing data | | 1.54 (1.34, 1.75)^‡^ | | 0.29 (0.14, 0.44)^‡^ | | 0.28 (0.13, 0.43)^‡^ |
| Days in LTCH Prior to Index Date | |  | | - | | - |
| 0 - 4 months | | Reference | | Reference | | Reference |
| > 4 months - 12   months | | -0.29 (-0.48, -0.11)^†^ | | -0.65 (-0.78, -0.52)^‡^ | | -0.65 (-0.78, -0.52)^‡^ |
| > 1 year - 2 years | | -0.01 (-0.19, 0.17) | | -0.75 (-0.89, -0.62)^‡^ | | -0.76 (-0.89, -0.62)^‡^ |
| > 2 years - 3 years | | 0.81 (0.59, 1.02)^‡^ | | -0.65 (-0.78, -0.51)^‡^ | | -0.65 (-0.78, -0.51)^‡^ |
| > 3 years | | 2.66 (2.50, 2.82)^‡^ | | -0.31 (-0.44, -0.17)^‡^ | | -0.30 (-0.44, -0.17)^‡^ |
| **Prevalent Geriatric Syndromes** |  | |  | |  | |
| Balance impairment | 10.48 (10.34, 10.60)^‡^ | | 5.69 (5.51, 5.87)^‡^ | | 5.66 (5.48, 5.84)^‡^ | |
| Bowel incontinence | 10.46 (10.37, 10.55)^‡^ | | 4.53 (4.38, 4.68)^‡^ | | 4.52 (4.37, 4.67)^‡^ | |
| Cognitive status |  | |  | |  | |
| Intact/borderline | Reference | | Reference | | Reference | |
| Mild/moderate   impairment | 3.89 (3.76, 4.01)^‡^ | | 1.67 (1.55, 1.79)^‡^ | | 1.66 (1.54, 1.78)^‡^ | |
| Moderate-   severe/severe   impairment | 11.73  (11.58, 11.87)^‡^ | | 5.27 (5.10, 5.44)^‡^ | | 5.26 (5.09, 5.43)^‡^ | |
| Hearing impairment |  | |  | |  | |
| None | Reference | | Reference | | Reference | |
| Hearing impaired | 1.73 (1.55, 1.90)^‡^ | | 0.03 (-0.08, 0.14) | | 0.03 (-0.08, 0.13) | |
| Missing data | -0.56 (-1.80, 0.67) | | 0.66 (-0.15, 1.46) | | 0.67 (-0.11, 1.46) | |
| Body mass index  (BMI) |  | |  | |  | |
| BMI < 18.5 | Reference | | Reference | | Reference | |
| 18.5 ≤ BMI ≤ 25 | -2.02 (-2.24, -1.80)^‡^ | | -0.54 (-0.68, -0.40)^‡^ | | -0.54 (-0.68, -0.40)^‡^ | |
| 25 < BMI <30 | -3.49 (-3.72, -3.26)^‡^ | | -0.87 (-1.03, -0.72)^‡^ | | -0.88 (-1.03, -0.72)^‡^ | |
| BMI ≥ 30 | -3.74 (-3.98, -3.50)^‡^ | | -0.59 (-0.75, -0.43)^‡^ | | -0.60 (-0.76, -0.44)^‡^ | |
| Pain |  | |  | |  | |
| None | Reference | | Reference | | Reference | |
| Less than daily   pain | -0.70 (-0.85, -0.56)^‡^ | | 0.29 (0.19, 0.39)^‡^ | | 0.29 (0.19, 0.39)^‡^ | |
| Daily or severe   daily pain | -0.12 (-0.29, 0.04) | | 0.82 (0.70, 0.94)^‡^ | | 0.83 (0.70, 0.95)^‡^ | |
| Pressure ulcer | 6.47 (6.23, 6.72)^‡^ | | 2.67 (2.52, 2.82)^‡^ | | 2.67 (2.52, 2.82)^‡^ | |
| Urinary  incontinence | 10.50  (10.40, 10.61)^‡^ | | 4.19 (4.04, 4.35)^‡^ | | 4.20 (4.04, 4.35)^‡^ | |
| Visual impairment |  | |  | |  | |
| None | Reference | | Reference | | Reference | |
| Moderate   impairment | 3.09 (2.97, 3.22)^‡^ | | 0.68 (0.59, 0.77)^‡^ | | 0.68 (0.59, 0.76)^‡^ | |
| Severe   impairment | 7.62 (7.40, 7.84)^‡^ | | 2.49 (2.33, 2.65)^‡^ | | 2.49 (2.33, 2.65)^‡^ | |
| **Prevalent Chronic Conditions** |  | |  | |  | |
| Arthritis | | -0.66 (-0.78, -0.54)^‡^ | | 0.08 (-.0003, 0.15) | | 0.08 (0.0003, 0.15)^*^ |
| Asthma | | -0.71 (-0.94, -0.48)^‡^ | | 0.10 (-0.04, 0.24) | | 0.10 (-0.04, 0.24) |
| Cancer | | -1.23 (-1.36, -1.11)^‡^ | | -0.12 (-0.19, -0.04)^†^ | | -0.12 (-0.19, -0.04)^†^ |
| Chronic kidney  disease | | 0.06 (0.08, 0.20)^‡^ | | 0.31 (0.22, 0.40)^‡^ | | 0.31 (0.22, 0.40)^‡^ |
| Coronary artery  disease | | -0.86 (-0.98, -0.74)^‡^ | | -0.13 (-0.21, -0.05)^†^ | | -0.13 (-0.21, -0.05)^†^ |
| Chronic obstructive  pulmonary disease | | -1.39 (-1.54, -1.25)^‡^ | | -0.07 ( -0.17, 0.02) | | -0.07 (-0.17, 0.02) |
| Dementia | | 3.39 (3.22, 3.55)^‡^ | | -0.22 (-0.35, -0.10)^†^ | | -0.23 (-0.36, -0.11)^‡^ |
| Diabetes | | -0.09 (-0.21, 0.04) | | -0.06 (-0.14, 0.02) | | -0.06 (-0.14, 0.02) |
| Epilepsy | | 2.17 (1.94, 2.41)^‡^ | | 0.47 (0.32, 0.61)^‡^ | | 0.47 (0.33, 0.62)^‡^ |
| Heart failure | | -0.24 (-0.38, -0.11)^‡^ | | 0.36 (0.27, 0.46)^‡^ | | 0.36 (0.27, 0.45)^‡^ |
| Limb paralysis or  amputation | | 4.49 (4.29, 4.70)^‡^ | | 1.78 (1.63, 1.93)^‡^ | | 1.77 (1.62, 1.92)^‡^ |
| Mood disorder | | 0.53 (0.41, 0.65)^‡^ | | 0.30 (0.22, 0.38)^‡^ | | 0.30 (0.22, 0.38)^‡^ |
| Parkinson’s disease | | 2.87 (2.66, 3.07)^‡^ | | 1.75 (1.63, 1.87)^‡^ | | 1.75 (1.63, 1.87)^‡^ |
| Peripheral vascular  disease | | -0.14 (-0.34, 0.07) | | 0.03 (-0.10, 0.16) | | 0.03 (-0.10, 0.16) |
| Psychiatric  conditions other  than depression and  dementia | | -1.35 (-1.48, -1.22)^‡^ | | -0.42 (-0.50, -0.33)^‡^ | | -0.42 (-0.50, -0.33)^‡^ |
| Stroke | | 1.85 (1.73, 1.98)^‡^ | | 0.46 (0.38, 0.55)^‡^ | | 0.46 (0.38, 0.55)^‡^ |
| **Fixed Long-Term Care Home Effects**^¶^ | | | | | | |
| LTCH Size | |  | |  |  | |
| Small (≤64) | | Reference | | N/A | | Reference |
| Medium (65 – 128) | | 0.10 (-0.38, 0.58) | | N/A | | -0.05 (-0.32, 0.21) |
| Large (129 – 192) | | 0.37 (-0.16, 0.89) | | N/A | | 0.08 (-0.24, 0.40) |
| Extra-large ( ≥193) | | 0.79 (0.19, 1.39) | | N/A | | 0.25 (-0.13, 0.63) |
| Ownership status | | | | | | |
| Not-for-profit | | Reference | | N/A | | Reference |
| For-profit | | 0.28 (-0.12, 0.68) | | N/A | | 0.23 (0.006, 0.46)^*^ |
| Missing data | | -3.64 (-5.26, -2.02)^‡^ | | N/A | | 0.44 (-0.47, 1.35) |
| Location | | | | | | |
| Rural | | Reference | | N/A | | Reference |
| Sub-urban | | 0.28 (-0.25, 0.82) | | N/A | | 0.14 (-0.22, 0.49) |
| Urban | | 1.47 (1.02, 1.92)^‡^ | | N/A | | -0.12 ( -0.41, 0.15) |
| Mean % residents received physio- or occupational therapy (Quartiles) | |  | |  | |  |
| Lowest quartile | | Reference | | N/A | | Reference |
| 2^nd^ quartile | | 0.004 (-0.57, 0.57) | | N/A | | 0.17 (-0.13, 0.47) |
| 3^rd^ quartile | | 0.78 (0.21, 1.35)^†^ | | N/A | | 0.14 (-0.17, 0.45) |
| Highest quartile | | 0.53 (-0.01, 1.07) | | N/A | | -0.05 (-0.35, 0.24) |
| Mean % residents restrained (Quartiles) | |  | |  | |  |
| Lowest quartile | | Reference | | N/A | | Reference |
| 2^nd^ quartile | | -0.32 (-0.89, 0.25) | | N/A | | 0.007 (-0.30, 0.32) |
| 3^rd^ quartile | | -0.19 (-0.79, 0.41) | | N/A | | -0.23 (-0.54, 0.07) |
| Highest quartile | | 0.50 (-0.06, 1.05) | | N/A | | -0.14 (-0.45, 0.16) |
| Median Resident ADL in each home (Quartiles) | |  | |  | |  |
| Lowest quartile | | Reference | | N/A | | Reference |
| 2^nd^ quartile | | 1.87 (1.58, 2.17) ^‡^ | | N/A | | 1.16 (0.87, 1.46) ^‡^ |
| 3^rd^ quartile | | 2.75 (2.45, 3.06) ^‡^ | | N/A | | 1.62 (1.30, 1.94) ^‡^ |
| Highest quartile | | 4.76 (4.43, 5.10) ^‡^ | | N/A | | 2.81 (2.50, 3.11) ^‡^ |
| **Random Effects** | |  | |  | |  |
| √ψ | | - | | 1.58 (1.50, 1.68) | | 1.21 (1.13, 1.28) |
| √θ | | - | | 4.90 (4.84, 4.96) | | 4.90 (4.84, 4.96) |
| **Derived Estimates** | |  | |  | |  |
| R^2^ | | - | | 0.627 | | 0.642 |
| ρ | | - | | 0.095 | | 0.057 |

Legend:

**Reference**: Variable category is the reference group for all other categories within that variable.

**^*^**p-value <0.05
**^†^**p-value <0.01

**^‡^**p-value <0.0001

^§^**Model 1:** Adjusted for resident age, sex, marital status, pre-admission neighborhood income quintile, number of days since admission to long-term care home; includes random intercept for long-term care homes

^||^**Model 2:** Adjusted for resident age, sex, marital status, pre-admission neighborhood income quintile, number of days since admission to long-term care home, as well as the following long-term care home variables: size, ownership type, location, proportion of residents who recently received physiotherapy or occupation therapy, proportion of residents restrained, and median resident disability. Also a random intercept for long-term care homes.

^¶^LTCH coefficient estimates have standard errors adjusted for clustering of residents within long-term care homes.

***√ψ***: Square root of between-long-term care home variance

***√θ***: Square root of within-long-term care home variance

The **null model** of disability containing only random LTCH intercepts and no explanatory resident or LTCH variables had a within-LTCH variance of 66.91 and a between-LTCH variance of 4.16; variances from all multivariable models were compared to these values to estimate proportion of variance explained (*R^2^*).
***R^2^***: The proportional reduction in the estimated total residual variance compared to the null model (Model 1)

**ρ**: Proportion of variance that is explained by LTCH characteristics = ψ/(ψ+θ)
**N/A**: Not applicable because variable not included in model.

## Table S6

*Model 1 Excluding Chronic Conditions, Geriatric Syndromes*

| **Variables** | **Model 1** | **Model 1 excluding chronic conditions** | **Model 1 excluding geriatric syndromes** | **Model 1, with only adjustment variables and select geriatric syndromes** |
| --- | --- | --- | --- | --- |
| **Resident Characteristics** |  |  |  |  |
| Age |  |  |  |  |
| 65 – 74 | Reference | Reference | Reference |  |
| 75 – 84 | 0.04 (-0.08, 0.17) | -0.08 (-0.21, 0.04) | 0.60 (0.39, 0.82)^‡^ | N/A |
| 85 – 94 | 0.18 (0.04, 0.31)^*^ | -0.10 (-0.24, 0.03) | 1.16 (0.93, 1.39)^‡^ | N/A |
| 95+ | 0.61 (0.42, 0.80)^‡^ | 0.20 (0.02, 0.39)^*^ | 2.61 (2.31, 2.91)^‡^ | N/A |
| Sex |  |  |  |  |
| Female | Reference | Reference | Reference |  |
| Male | -0.37 (-0.46, -0.28) ^‡^ | -0.18 (-0.27, -0.09)^‡^ | -1.38 (-1.53, -1.24)^‡^ | N/A |
| Marital Status |  |  |  |  |
| Married | Reference | Reference | Reference |  |
| Widowed | -0.41 (-0.50, -0.31)^‡^ | -0.47 (-0.56, -0.37)^‡^ | -1.58 (-1.74, -1.43)^‡^ | N/A |
| Never married,   separated or   divorced | -0.60 (-0.73, -0.48)^‡^ | -0.75 (-0.87, -0.62)^‡^ | -2.22 (-2.42, -2.02)^‡^ | N/A |
| Missing data on   marital status | -0.64 (-0.96, -0.32)^‡^ | -0.76 (-1.10, -0.43)^‡^ | -1.45 (-1.94, -0.96)^‡^ | N/A |
| Pre-LTCH Neighborhood Income Quintile |  |  |  |  |
| 1 (low) | Reference | Reference | Reference |  |
| 2 | 0.13 (0.01, 0.26)^*^ | 0.16 (0.03, 0.28)^*^ | 0.29 (0.10, 0.48)^†^ | N/A |
| 3 | 0.19 (0.07, 0.31)^†^ | 0.22 (0.10, 0.34)^‡^ | 0.37 (0.17, 0.57)^‡^ | N/A |
| 4 | 0.29 (0.17, 0.41)^‡^ | 0.32 (0.19, 0.44)^‡^ | 0.61 (0.41, 0.80)^‡^ | N/A |
| 5 (high) | 0.23 (0.10, 0.37)^†^ | 0.27 ( 0.13, 0.41)^‡^ | 0.59 (0.39, 0.79)^‡^ | N/A |
| Missing data | 0.29 (0.14, 0.44)^‡^ | 0.34 (0.18, 0.49)^‡^ | 1.07 (0.81, 1.32)^‡^ | N/A |
| Days in LTC Prior to Index Date | - |  |  |  |
| 0 - 4 months | Reference | Reference | Reference |  |
| > 4 months - 12   months | -0.65 (-0.78, -0.52)^‡^ | -0.62 (-0.75, -0.48)^‡^ | -0.48 (-0.66, -0.30)^‡^ | N/A |
| > 1 year - 2 years | -0.75 (-0.89, -0.62)^‡^ | 0.73 (-0.87, -0.60)^‡^ | -0.38 (-0.58, -0.17)^‡^ | N/A |
| > 2 years - 3 years | -0.65 (-0.78, -0.51)^‡^ | -0.64 (-0.78, -0.50)^‡^ | 0.32 (0.08, 0.57)^‡^ | N/A |
| > 3 years | -0.31 (-0.44, -0.17)^‡^ | -0.28 (-0.41, -0.14)^‡^ | 2.10 (1.88, 2.33)^*^ | N/A |
| **Prevalent Geriatric Syndromes** |  |  |  |  |
| Balance impairment | 5.69 (5.51, 5.87)^‡^ | 6.04 (5.86, 6.22)^‡^ | N/A | 6.38 (6.19, 6.56) ^‡^ |
| Bowel incontinence | 4.53 (4.38, 4.68)^‡^ | 4.61 (4.46, 4.77)^‡^ | N/A | 4.91 (4.75, 5.07)^‡^ |
| Cognitive status |  |  |  |  |
| Intact/borderline | Reference | Reference |  |  |
| Mild/moderate   impairment | 1.67 (1.55, 1.79)^‡^ | 1.51 (1.39, 1.63)^‡^ | N/A | 1.46 (1.34, 1.59)^‡^ |
| Moderate-   severe/severe   impairment | 5.27 (5.10, 5.44)^‡^ | 4.97 (4.80, 5.13)^‡^ | N/A | 5.27 (5.11, 5.44)^‡^ |
| Hearing impairment |  |  |  |  |
| None | Reference | Reference |  |  |
| Hearing impaired | 0.03 (-0.08, 0.14) | -0.01 (-0.12, 0.10) | N/A | N/A |
| Missing data | 0.66 (-0.15, 1.46) | 0.62 (-0.18, 1.42) | N/A | N/A |
| Body mass index   (BMI) |  |  |  |  |
| BMI < 18.5 | Reference | Reference |  |  |
| 18.5 ≤ BMI ≤ 25 | -0.54 (-0.68, -0.40)^‡^ | -0.53 (-0.67, -0.39)^‡^ | N/A | N/A |
| 25 < BMI <30 | -0.87 (-1.03, -0.72)^‡^ | -0.86 (-1.01, -0.70)^‡^ | N/A | N/A |
| BMI ≥ 30 | -0.59 (-0.75, -0.43)^‡^ | -0.56 (-0.72, -0.41)^‡^ | N/A | N/A |
| Pain |  |  |  |  |
| None | Reference | Reference |  |  |
| Less than daily   pain | 0.29 (0.19, 0.39)^‡^ | 0.35 (0.25, 0.45)^‡^ | N/A | N/A |
| Daily or severe   daily pain | 0.82 (0.70, 0.94)^‡^ | 0.86 (0.74, 0.98)^‡^ | N/A | N/A |
| Pressure ulcer | 2.67 (2.52, 2.82)^‡^ | 2.79 (2.64, 2.94)^‡^ | N/A | N/A |
| Urinary   incontinence | 4.19 (4.04, 4.35)^‡^ | 4.32 (4.16, 4.48)^‡^ | N/A | 4.26 (4.09, 4.42)^‡^ |
| Visual impairment |  |  |  |  |
| None | Reference | Reference |  |  |
| Moderate   impairment | 0.68 (0.59, 0.77)^‡^ | 0.72 (0.63, 0.81)^‡^ | N/A | N/A |
| Severe impairment | 2.49 (2.33, 2.65)^‡^ | 2.47 (2.31, 2.63)^‡^ | N/A | N/A |
| **Prevalent Chronic Conditions** |  |  |  |  |
| Arthritis | 0.08 (-.0003, 0.15) | N/A | -0.26 (-0.37, -0.14)^‡^ | N/A |
| Asthma | 0.10 (-0.04, 0.24) | N/A | -0.16 (-0.38, 0.06) | N/A |
| Cancer | -0.12 (-0.19, -0.04)^†^ | N/A | -0.80 (-0.92, -0.68)^‡^ | N/A |
| Chronic kidney   disease | 0 .31 (0.22, 0.40)^‡^ | N/A | 0.52 (0.39, 0.65)^‡^ | N/A |
| Coronary artery   disease | -0.13 (-0.21, -0.05)^†^ | N/A | -0.61 (-0.74, -0.48)^‡^ | N/A |
| Chronic obstructive   pulmonary disease | -0.07 ( -0.17, 0.02) | N/A | -0.61 (-0.76, -0.45)^‡^ | N/A |
| Dementia | -0.22 (-0.35, -0.10)^†^ | N/A | 3.29 (3.07, 3.50)^‡^ | N/A |
| Diabetes | -0.06 (-0.14, 0.02) | N/A | -0.03 (-0.15, 0.10) | N/A |
| Epilepsy | 0.47 (0.32, 0.61)^‡^ | N/A | 1.71 (1.48, 1.94)^‡^ | N/A |
| Heart failure | 0.36 (0.27, 0.46)^‡^ | N/A | 0.45 (0.32, 0.58)^‡^ | N/A |
| Limb paralysis or   amputation | 1.78 (1.63, 1.93)^‡^ | N/A | 4.02 (3.79, 4.24)^‡^ | N/A |
| Mood disorder | 0.30 (0.22, 0.38)^‡^ | N/A | 0.38 (0.26, 0.51)^‡^ | N/A |
| Parkinson’s disease | 1.75 (1.63, 1.87)^‡^ | N/A | 3.11 (2.94, 3.29)^‡^ | N/A |
| Peripheral vascular   disease | 0.03 (-0.10, 0.16) | N/A | 0.09 (-0.11, 0.29) | N/A |
| Psychiatric   conditions other   than depression and   dementia | -0.42 (-0.50, -0.33)^‡^ | N/A | -1.09 (-1.23, -0.95)^‡^ | N/A |
| Stroke | 0.46 (0.38, 0.55)^‡^ | N/A | 1.21 (1.07, 1.34)^‡^ | N/A |
| **Random Effects** |  |  |  |  |
| √ψ | 1.58 | 1.62 | 1.85 | 1.61 |
| √θ | 4.90 | 4.97 | 7.73 | 5.09 |
| **Derived Estimates** |  |  |  |  |
| R^2^ | 0.627 | 0.616 | 0.112 | 0.599 |
| ρ | 0.095 | 0.096 | 0.054 | 0.091 |

Legend:

**Model 1:** Adjusted for resident age, sex, marital status, pre-admission neighborhood income quintile, number of days since admission to long-term care home; includes random intercept for long-term care homes

**Reference**: Variable category is the reference group for all other categories within that variable.

**^*^**p-value <0.05
**^†^**p-value <0.01

**^‡^**p-value <0.0001
***√ψ***: Square root of between-long-term care home variance

***√θ***: Square root of within-long-term care home variance

The **null model** of disability containing only random LTCH intercepts and no explanatory resident or LTCH variables had a within-LTCH variance of 66.91 and a between-LTCH variance of 4.16; variances from all multivariable models were compared to these values to estimate proportion of variance explained (*R^2^*).
***R^2^***: The proportional reduction in the estimated total residual variance compared to the null model (Model 1)

**ρ**: Proportion of variance that is explained by LTCH characteristics = ψ/(ψ+θ)
**N/A**: Not applicable because variable not included in model.

## Table S7

*All Variable Coefficient Estimates from Stratified Versions of Model 1*

|  | **Model 1** | **Sex Stratified Models** | | **Age Stratified Models** | | | | **Cognitive Status-Stratified Models** | |
| --- | --- | --- | --- | --- | --- | --- | --- | --- | --- |
|  |  | **Females**  **(n=54,953)** | **Males  (n=22,212)** | **Age 65-74 (n=7,859)** | **Age 75-84 (n=25,703)** | **Age 85-94 (n=36,676)** | **Age 95-105**  **(n=6,927)** | **No cognitive impairment**  **(n=18,426)** | **Cognitive impairment present**  **(n = 58,739)** |
| Age |  |  |  |  |  |  |  |  |  |
| 65 – 74 | Reference | Reference | Reference | Reference | Reference | Reference | Reference | Reference | Reference |
| 75 – 84 | 0.04  (-0.08, 0.17) | 0.08  (-0.009, 0.25) | -0.04  (-0.25, 0.16) | N/A | N/A | N/A | N/A | -0.15  (-0.40, 0.10) | 0.04  (-0.12, 0.20) |
| 85 – 94 | 0.18  (0.04, 0.31)^*^ | 0.23  (0.05, 0.41)^*^ | 0.04  (-0.18, 0.26) | N/A | N/A | N/A | N/A | -0.10  (-0.39, 0.18) | -0.01  (-0.18, 0.16) |
| 95+ | 0.61  (0.42, 0.80)^‡^ | 0.69  (0.46, 0.91)^‡^ | 0.36  (-0.02, 0.74) | N/A | N/A | N/A | N/A | 0.62  (0.21, 1.02)^†^ | 0.16  (-0.06, 0.38) |
| Sex |  |  |  |  |  |  |  |  |  |
| Female | Reference | Reference | Reference | Reference | Reference | Reference | Reference | Reference | Reference |
| Male | -0.37  (-0.46, -0.28) ^‡^ | N/A | N/A | -0.50  (-0.75, -0.26)^‡^ | -0.40  (-0.54, -0.26)^‡^ | -0.28  (-0.43, -0.13)^‡^ | -0.44  (-0.79, -0.09)^*^ | -0.29  (-0.48, -0.10)^†^ | -0.47  (-0.58, -0.36)^‡^ |
| Marital Status |  |  |  |  |  |  |  |  |  |
| Married | Reference | Reference | Reference | Reference | Reference | Reference | Reference | Reference | Reference |
| Widowed | -0.41  (-0.50, -0.31)^‡^ | -0.45  (-0.57, -0.34)^‡^ | -0.28  (-0.44, -0.12)^†^ | -0.61  (-0.93, -0.28)^‡^ | -0.46  (-0.61, -0.31)^‡^ | -0.25  (-0.40, -0.11)^†^ | -0.26  (-0.77, 0.24) | -0.23  (-0.43, -0.02)^*^ | -0.71  (-0.82, -0.60)^‡^ |
| Never married,   separated or   divorced | -0.60  (-0.73, -0.48)^‡^ | -0.59  (-0.76, -0.43)^‡^ | -0.60  (-0.79, -0.41)^‡^ | -0.81  (-1.09, -0.52)^‡^ | -0.75  (-0.95, -0.55)^‡^ | -0.30  (-0.51, -0.08)^†^ | -0.15  (-0.80, 0.50) | -0.33  (-0.59, -0.07)^*^ | -1.09  (-1.23, -0.94)^‡^ |
| Missing data on   marital status | -0.64  (-0.96, -0.32)^‡^ | -0.63  (-1.06, -0.20)^†^ | -0.64  (-1.13, -0.15)^*^ | -0.82  (-1.70, 0.06) | -0.72  (-1.30, -0.13)^*^ | -0.47  (-1.01, 0.06) | -0.67  (-1.84, 0.51) | -0.08  (-0.85, 0.68) | -0.97  (-1.36, -0.57)^‡^ |
| Pre-LTCH Neighborhood Income Quintile |  |  |  |  |  |  |  |  |  |
| 1 (low) | Reference | Reference | Reference | Reference | Reference | Reference | Reference | Reference | Reference |
| 2 | 0.13  (0.01, 0.26)^*^ | 0.13  (-0.01, 0.28) | 0.16  (-0.06, 0.38) | 0.15  (-0.20, 0.51) | -0.01  (-0.21, 0.19) | 0.24  (0.08, 0.41)^†^ | 0.18  (-0.19, 0.56) | 0.14  (-0.13, 0.41) | 0.15  (0.006, 0.29)^*^ |
| 3 | 0.19  (0.07, 0.31)^†^ | 0.21  (0.07, 0.35)^†^ | 0.12  (-0.08, 0.31) | 0.08  (-0.26, 0.42) | 0.08  (-0.13, 0.29) | 0.27  (0.11, 0.43)^†^ | 0.17  (-0.20, 0.54) | 0.17  (-0.11, 0.45) | 0.19  (0.05, 0.32)^†^ |
| 4 | 0.29  (0.17, 0.41)^‡^ | 0.32  (0.17, 0.47)^‡^ | 0.23  (0.03, 0.44)^*^ | 0.36  (-0.03, 0.76) | 0.20  (-0.007, 0.40) | 0.36  (0.19, 0.54)^‡^ | 0.27  (-0.12, 0.66) | 0.34  (0.06, 0.61)^*^ | 0.30  (0.16, 0.44)^‡^ |
| 5 (high) | 0.23  (0.10, 0.37)^†^ | 0.27  (0.11, 0.42)^†^ | 0.18  (-0.05, 0.40) | 0.34  (-0.07, 0.76) | 0.25  (0.03, 0.46)^*^ | 0.24  (0.05, 0.43)^†^ | 0.05  (-0.35, 0.46) | 0.14  (-0.16, 0.45) | 0.32  (0.17, 0.47)^‡^ |
| Missing data | 0.29  (0.14, 0.44)^‡^ | 0.27  (0.09, 0.45)^†^ | 0.40  (0.15, 0.65)^†^ | 0.009  (-0.43, 0.45) | 0.17  (-0.05, 0.40) | 0.46  (0.25, 0.67)^‡^ | 0.21  (-0.22, 0.64) | -0.07  (-0.40, 0.27) | 0.44  (0.27, 0.61)^‡^ |
| Days in LTC Prior to Index Date |  |  |  |  |  |  |  |  |  |
| 0 - 4 months | Reference | Reference | Reference | Reference | Reference | Reference | Reference | Reference | Reference |
| > 4 months - 12   months | -0.65  (-0.78, -0.52)^‡^ | -0.64  (-0.79, -0.48)^‡^ | -0.66  (-0.86, -0.45)^‡^ | -0.36  (-0.70, -0.02)^*^ | -0.61  (-0.81, -0.41)^‡^ | -0.70  (-0.86, -0.53)^‡^ | -0.91  (-1.35, -0.48)^‡^ | -1.17  (-1.42, -0.93)^‡^ | -0.44  (-0.58, -0.29)^‡^ |
| > 1 year - 2   years | -0.75  (-0.89, -0.62)^‡^ | -0.77  (-0.93, -0.61)^‡^ | -0.68  (-0.89, -0.48)^‡^ | -0.30  (-0.67, 0.06) | -0.64  (-0.86, -0.43)^‡^ | -0.86  (-1.04, -0.69)^‡^ | -1.10  (-1.53, -0.68)^‡^ | -1.59  (-1.85, -1.32)^‡^ | -0.36  (-0.51, -0.21)^‡^ |
| > 2 years - 3   years | -0.65  (-0.78, -0.51)^‡^ | -0.63  (-0.80, -0.46)^‡^ | -0.63  (-0.88, -0.38)^‡^ | -0.41  (-0.85, 0.02) | -0.42  (-0.65, -0.19)^‡^ | -0.83  (-1.02, -0.64)^‡^ | -0.61  (-1.06, -0.17)^†^ | -1.76  (-2.05, -1.47)^‡^ | -0.09  (-0.25, 0.07) |
| > 3 years | -0.31  (-0.44, -0.17)^‡^ | -0.21  (-0.36, -0.05)^‡^ | -0.61  (-0.81, -0.41)^‡^ | -0.40  (-0.75, -0.05)^*^ | -0.15  (-0.36, 0.06) | -0.37  (-0.53, -0.21)^‡^ | -0.56  (-0.92, -0.20)^†^ | -1.96  (-2.20, -1.71)^‡^ | 0.64  (0.48, 0.79)^‡^ |
| Prevalent Geriatric Syndromes |  |  |  |  |  |  |  |  |  |
| Balance   impairment | 5.69  (5.51, 5.87)^‡^ | 5.73  (5.52, 5.93)^‡^ | 5.51  (5.28, 5.74)^‡^ | 5.94  (5.57, 6.31)^‡^ | 5.71  (5.49, 5.93)^‡^ | 5.46  (5.25, 5.68)^‡^ | 5.42  (5.00, 5.85)^‡^ | 5.55  (5.31, 5.80)^‡^ | 5.95  (5.75, 6.16)^‡^ |
| Bowel   incontinence | 4.53  (4.38, 4.68)^‡^ | 4.43  (4.26, 4.60)^‡^ | 4.77  (4.57, 4.97)^‡^ | 5.00  (4.66, 5.33)^‡^ | 4.61  (4.41, 4.82)^‡^ | 4.46  (4.28, 4.65)^‡^ | 3.98  (3.65, 4.32)^‡^ | 4.60  (4.35, 4.86)^‡^ | 5.43  (5.26, 5.60)^‡^ |
| Cognitive status |  |  |  |  |  |  |  |  |  |
| Intact/borderline | Reference | Reference | Reference | Reference | Reference | Reference | Reference | Reference | Reference |
| Mild/moderate   impairment | 1.67  (1.55, 1.79)^‡^ | 1.69  (1.55, 1.83)^‡^ | 1.67  (1.46, 1.88)^‡^ | 1.14  (0.85, 1.43)^‡^ | 1.51  (1.31, 1.70)^‡^ | 1.88  (1.71, 2.04)^‡^ | 1.86  (1.50, 2.22)^‡^ | N/A | N/A |
| Moderate-   severe/severe   impairment | 5.27  (5.10, 5.44)^‡^ | 5.40  (5.21, 5.59)^‡^ | 4.94  (4.67, 5.21)^‡^ | 4.21  (3.81, 4.61)^‡^ | 5.16  (4.91, 5.41)^‡^ | 5.57  (5.34, 5.79)^‡^ | 5.35  (4.88, 5.81)^‡^ | N/A | N/A |
| Hearing   impairment |  |  |  |  |  |  |  |  |  |
| None | Reference | Reference | Reference | Reference | Reference | Reference | Reference | Reference | Reference |
| Hearing   impaired | 0.03  (-0.08, 0.14) | 0.02  (-0.12 0.15) | 0.07  (-0.12, 0.26) | -0.13  (-0.71, 0.45) | -0.04  (-0.26, 0.18) | -0.04  (-0.18, 0.11) | 0.26  (0.002, 0.51)^*^ | 0.43  (0.14, 0.72)^†^ | 0.08  (-0.04, 0.20) |
| Missing data | 0.66  (-0.15, 1.46) | 0.33  (-0.67, 1.34) | 1.21  (-0.09, 2.52) | 1.85  (-0.79, 4.49) | 0.005  (-1.21, 1.22) | 1.18  (-0.21, 2.57) | 0.007  (-3.06, 3.08) | 0.32  (-1.27, 1.90) | 0.74  (-0.16, 1.64) |
| Body mass   index (BMI) |  |  |  |  |  |  |  |  |  |
| BMI < 18.5 | Reference | Reference | Reference | Reference | Reference | Reference | Reference | Reference | Reference |
| 18.5 ≤ BMI ≤   25 | -0.54  (-0.68, -0.40)^‡^ | -0.56  (-0.71, -0.41)^‡^ | -0.52  (-0.84, -0.21)^†^ | -0.03  (-0.57, 0.52) | -0.46  (-0.72, -0.19)^†^ | -0.67  (-0.86, -0.49)^‡^ | -0.55  (-0.89, -0.20)^†^ | -0.80  (-1.16, -0.43)^‡^ | -0.51  (-0.66, -0.36)^‡^ |
| 25 < BMI <30 | -0.87  (-1.03, -0.72)^‡^ | -0.83  (-0.99, -0.66)^‡^ | -1.04  (-1.36, -0.71)^‡^ | -0.69  (-1.24, -0.14)^*^ | -0.82  (-1.09, -0.54)^‡^ | -0.96  (-1.17, -0.76)^‡^ | -0.83  (-1.23, -0.44)^‡^ | -1.04  (-1.42, -0.67)^‡^ | -0.97  (-1.14, -0.80)^‡^ |
| BMI ≥ 30 | -0.59  (-0.75, -0.43)^‡^ | -0.52  (-0.69, -0.34)^‡^ | -0.89  (-1.24, -0.53)^‡^ | -0.68  (-1.23, -0.12)^*^ | -0.55  (-0.84, -0.26)^‡^ | -0.62  (-0.84, -0.41)^‡^ | -0.31  (-0.80, 0.18) | -0.50  (-0.89,-0.10)^†^ | -0.98  (-1.16, -0.81)^‡^ |
| Pain |  |  |  |  |  |  |  |  |  |
| None | Reference | Reference | Reference | Reference | Reference | Reference | Reference | Reference | Reference |
| Less than   daily pain | 0.29  (0.19, 0.39)^‡^ | 0.25  (0.13, 0.36)^‡^ | 0.39  (0.22, 0.57)^‡^ | 0.26  (-0.03, 0.54) | 0.18  (0.03, 0.34)^*^ | 0.34  (0.20, 0.48)^‡^ | 0.19  (-0.09, 0.48) | 0.50  (0.30, 0.69)^‡^ | -0.007  (-0.12, 0.11) |
| Daily or   severe daily   pain | 0.82  (0.70, 0.94)^‡^ | 0.78  (0.64, 0.92)^‡^ | 0.90  (0.68, 1.13)^‡^ | 0.70  (0.35, 1.05)^‡^ | 0.76  (0.57, 0.95)^‡^ | 0.86  (0.69, 1.02)^‡^ | 0.67  (0.28, 1.05)^†^ | 0.81  (0.59, 1.04)^‡^ | 0.62  (0.47, 0.76)^‡^ |
| Pressure ulcer | 2.67  (2.52, 2.82)^‡^ | 2.70  (2.52, 2.87)^‡^ | 2.59  (2.32, 2.86)^‡^ | 3.03  (2.57, 3.48)^‡^ | 2.70  (2.45, 2.95)^‡^ | 2.63  (2.42, 2.84)^‡^ | 2.39  (1.98, 2.81)^‡^ | 3.34  (2.98, 3.71)^‡^ | 2.78  (2.62, 2.94)^‡^ |
| Urinary   incontinence | 4.19  (4.04, 4.35)^‡^ | 4.30  (4.12, 4.49)^‡^ | 3.97  (3.76, 4.19)^‡^ | 4.00  (3.66, 4.34)^‡^ | 4.19  (3.98, 4.40)^‡^ | 4.28  (4.08, 4.49)^‡^ | 4.06  (3.66, 4.45)^‡^ | 4.48  (4.25, 4.71)^‡^ | 4.28  (4.10, 4.46)^‡^ |
| Visual   impairment |  |  |  |  |  |  |  |  |  |
| None | Reference | Reference | Reference | Reference | Reference | Reference | Reference | Reference | Reference |
| Moderate   impairment | 0.68  (0.59, 0.77)^‡^ | 0.68  (0.57, 0.78)^‡^ | 0.70  (0.55, 0.85)^‡^ | 0.73  (0.48, 0.98)^‡^ | 0.64  (0.50, 0.79)^‡^ | 0.67  (0.55, 0.79)^‡^ | 0.80  (0.53, 1.07)^‡^ | 0.53  (0.33, 0.73)^‡^ | 0.99  (0.88, 1.09)^‡^ |
| Severe   impairment | 2.49  (2.33, 2.65)^‡^ | 2.45  (2.27, 2.63)^‡^ | 2.58  (2.30, 2.86)^‡^ | 2.82  (2.38, 3.26)^‡^ | 2.72  (2.45, 3.00)^‡^ | 2.39  (2.18, 2.60)^‡^ | 2.21  (1.83, 2.59)^‡^ | 1.98  (1.49, 2.48)^‡^ | 3.50  (3.32, 3.67)^‡^ |
| Prevalent Chronic Conditions |  |  |  |  |  |  |  |  |  |
| Arthritis | 0.08  (-.0003, 0.15) | 0.09  (-0.007, 0.18) | 0.04  (-0.09, 0.16) | -0.16  (-0.40, 0.08) | 0.10  (-0.03, 0.23) | 0.12  (0.006, 0.23)^*^ | 0.13  (-0.11, 0.38) | 0.13  (-0.05, 0.32) | -0.10 (-0.18,  -0.008)^*^ |
| Asthma | 0.10  (-0.04, 0.24) | 0.09  (-0.06, 0.24) | 0.17  (-0.11, 0.45) | 0.03  (-0.42, 0.48) | 0.10  (-0.14, 0.34) | 0.20  (-0.001, 0.40) | -0.11  (-0.62, 0.39) | 0.07  (-0.22, 0.35) | 0.08  (-0.07, 0.24) |
| Cancer | -0.12  (-0.19, -0.04)^†^ | -0.15  (-0.25, -0.06)^†^ | -0.06  (-0.20, 0.06) | -0.09  (-0.36, 0.18) | -0.16  (-0.29, -0.03)^*^ | -0.08  (-0.20, 0.02) | -0.17  (-0.43, 0.08) | -0.20  (-0.38, -0.03)^*^ | -0.21  (-0.30, -0.12)^‡^ |
| Chronic kidney   disease | 0 31  (0.22, 0.40)^‡^ | 0.26  (0.15, 0.37)^‡^ | 0.40  (0.24, 0.55)^‡^ | 0.39  (0.09, 0.68)^*^ | 0.32  (0.17, 0.48)^‡^ | 0.31  (0.18, 0.44)^‡^ | 0.32  (0.02, 0.62)^*^ | 0.22  (0.03, 0.41)^*^ | 0.26,  (0.15, 0.36)^‡^ |
| Coronary artery   disease | -0.13  (-0.21, -0.05)^†^ | -0.11  (-0.20, -0.02)^*^ | -0.18  (-0.32, -0.04)^*^ | -0.29  (-0.56, -0.02)^*^ | -0.04  (-0.17, 0.10) | -0.15  (-0.26, -0.04)^*^ | -0.19  (-0.44, 0.05) | -0.25  (-0.43, -0.08)^†^ | -0.17  (-0.26, -0.07)^‡^ |
| Chronic   obstructive   pulmonary  disease | -0.07  (-0.17, 0.02) | -0.09  (-0.21, 0.02) | -0.04  (-0.20, 0.12) | -0.09  (-0.38, 0.21) | -0.30  (-0.47, -0.14)^‡^ | 0.08  (-0.05, 0.21) | 0.11  (-0.21, 0.44) | -0.16  (-0.35, 0.03) | -0.23  (-0.34, -0.12)^‡^ |
| Dementia | -0.22  (-0.35, -0.10)^†^ | -0.32  (-0.47, -0.17)^‡^ | 0.02  (-0.20, 0.23) | -0.41  (-0.72, -0.10)^*^ | -0.39  (-0.61, -0.17)^‡^ | -0.10  (-0.29, 0.08) | 0.21  (-0.16, 0.59) | -0.25  (-0.43, -0.06)^†^ | 0.23  (0.06, 0.40)^†^ |
| Diabetes | -0.06  (-0.14, 0.02) | -0.03  (-0.13, 0.07) | -0.12  (-0.25, 0.01) | -0.24  (-0.49, 0.009) | -0.10  (-0.23, 0.03) | -0.004  (-0.12, 0.12) | 0.32  (0.04, 0.60)^*^ | -0.34  (-0.52, -0.16)^‡^ | -0.12  (-0.21, -0.03)^*^ |
| Epilepsy | 0.47  (0.32, 0.61)^‡^ | 0.61  (0.44, 0.78)^‡^ | 0.20  (-0.03, 0.44) | 0.47  (0.16, 0.77)^†^ | 0.36  (0.14, 0.58)^†^ | 0.60  (0.37, 0.84)^‡^ | -0.05  (-0.77, 0.67) | 0.13  (-0.23, 0.49) | 0.62  (0.46, 0.79)^‡^ |
| Heart failure | 0.36  (0.27, 0.46)^‡^ | 0.36  (0.25, 0.47)^‡^ | 0.37  (0.20, 0.53)^‡^ | 0.31  (-0.009, 0.63) | 0.41  (0.24, 0.58)^‡^ | 0.35  (0.22, 0.48)^‡^ | 0.34  (0.06, 0.61)^*^ | 0.47  (0.29, 0.65)^‡^ | 0.20  (0.09, 0.31)^‡^ |
| Limb paralysis   or   amputation | 1.78  (1.63, 1.93)^‡^ | 1.81  (1.63, 2.00)^‡^ | 1.79  (1.55, 2.02)^‡^ | 1.59  (1.27, 1.91)^‡^ | 1.93  (1.70, 2.17)^‡^ | 1.77  (1.55, 1.99)^‡^ | 1.57  (1.11, 2.04)^‡^ | 2.54  (2.24, 2.83)^‡^ | 1.44  (1.27, 1.60)^‡^ |
| Mood disorder | 0.30  (0.22, 0.38)^‡^ | 0.32  (0.23, 0.42)^‡^ | 0.26  (0.12, 0.41)^‡^ | -0.10  (-0.33, 0.14) | 0.20  (0.08, 0.33)^†^ | 0.44  (0.33, 0.55)^‡^ | 0.38  (0.12, 0.64)^†^ | 0.42  (0.23, 0.60)^‡^ | 0.15  (0.06, 0.24)^†^ |
| Parkinson’s   disease | 1.75  (1.63, 1.87)^‡^ | 1.79  (1.63, 1.95)^‡^ | 1.72  (1.53, 1.91)^‡^ | 1.61  (1.25, 1.97)^‡^ | 1.87  (1.69, 2.06)^‡^ | 1.66  (1.46, 1.86)^‡^ | 1.66  (1.09, 2.23)^‡^ | 2.18  (1.89, 2.47)^‡^ | 1.54  (1.41, 1.67)^‡^ |
| Peripheral   vascular   disease | 0.03  (-0.10, 0.16) | 0.11  (-0.05, 0.27) | -0.10  (-0.31, 0.11) | -0.24  (-0.65, 0.17) | 0.01  (-0.21, 0.23) | 0.13  (-0.06, 0.31) | 0.001  (-0.44, 0.45) | -0.18  (-0.43, 0.06) | -0.03  (-0.18, 0.13) |
| Psychiatric   conditions other   than depression   and   dementia | -0.42  (-0.50, -0.33)^‡^ | -0.39  (-0.49, -0.29)^‡^ | -0.46  (-0.62, -0.31)^‡^ | -0.61  (-0.84, -0.39)^‡^ | -0.38  (-0.52, -0.24)^‡^ | -0.35  (-0.48, -0.23)^‡^ | -0.20  (-0.49, 0.09) | -0.65  (-0.83, -0.46)^‡^ | -0.39  (-0.48, -0.30)^‡^ |
| Stroke | 0.46  (0.38, 0.55)^‡^ | 0.48  (0.37, 0.58)^‡^ | 0.45  (0.29, 0.60)^‡^ | 0.39  (0.13, 0.66)^†^ | 0.48  (0.34, 0.63)^‡^ | 0.45  (0.32, 0.58)^‡^ | 0.56  (0.28, 0.83)^‡^ | 0.68  (0.49, 0.86)^‡^ | 0.35  (0.25, 0.44)^‡^ |

Legend:

**Model 1:** Adjusted for resident age, sex, marital status, pre-admission neighborhood income quintile, number of days since admission to long-term care home; includes random intercept for long-term care homes

**Reference**: Variable category is the reference group for all other categories within that variable.

**^*^**p-value <0.05
**^†^**p-value <0.01

**^‡^**p-value <0.0001
**N/A** – Not applicable; variable not included in indicated model.

## Table S8

*Sensitivity of Model 1 Findings to Unmeasured LTCH Variables and Lack of adjustment for Long-Term Care Homes*

| **Variables** | **Model 1** | **Model 1 with fixed effect for LTCHs** | **Model 1 with no Random Effects for LTCHs** |
| --- | --- | --- | --- |
|  | Estimate (95% CI) | Estimate (95% CI) | Estimate (95% CI) |
| **Resident Characteristics** |  |  |  |
| Age |  |  |  |
| 65 – 74 | Reference | Reference | Reference |
| 75 – 84 | 0.04 (-0.08, 0.17) | 0.04 (-0.09, 0.17) | 0.12 (-0.01, 0.25) |
| 85 – 94 | 0.18 (0.04, 0.31)^*^ | 0.17 (0.04, 0.31)^*^ | 0.27 (0.12, 0.42)^‡^ |
| 95+ | 0.61 (0.42, 0.80)^‡^ | 0.60 (0.42, 0.78)^‡^ | 0.76 (0.55, 0.97)^‡^ |
| Sex |  |  |  |
| Female | Reference | Reference | Reference |
| Male | -0.37 (-0.46, -0.28)^‡^ | -0.36 ( -0.45, -0.28)^‡^ | -0.40 (-0.50, -0.30)^‡^ |
| Marital Status |  |  |  |
| Married | Reference | Reference | Reference |
| Widowed | -0.41 (-0.50, -0.31)^‡^ | -0.41 (-0.50, -0.31)^‡^ | -0.38 (-0.49, -0.27)^‡^ |
| Never married,   separated or   divorced | -0.60 (-0.73, -0.48)^‡^ | -0.61 (-0.73, -0.49)^‡^ | -0.55 (-0.70, -0.41)^‡^ |
| Missing data on   marital status | -0.64 (-0.96, -0.32)^‡^ | -0.64 (-0.96, -0.32)^‡^ | -0.64 (-1.08, -0.19)^†^ |
| Pre-LTCH Neighborhood Income Quintile |  |  |  |
| 1 (low) | Reference | Reference | Reference |
| 2 | 0.13 (0.01, 0.26)^*^ | 0.13 (0.02, 0.25)^*^ | 0.14 (-0.02, 0.30) |
| 3 | 0.19 (0.07, 0.31)^†^ | 0.20 (0.08, 0.31)^†^ | 0.10 (-0.07, 0.28) |
| 4 | 0.29 (0.17, 0.41)^‡^ | 0.28 (0.16, 0.40)^‡^ | 0.30 (0.13, 0.48)^†^ |
| 5 (high) | 0.23 (0.10, 0.37)^†^ | 0.23 (0.11, 0.35)^‡^ | 0.25 (0.05, 0.45)^*^ |
| Missing data | 0.29 (0.14, 0.44)^‡^ | 0.29 (0.14, 0.43)^‡^ | 0.23 (-0.02, 0.48) |
| Days in LTC Prior to Index Date | - |  |  |
| 0 - 4 months | Reference | Reference | Reference |
| > 4 months - 12   months | -0.65 (-0.78, -0.52)^‡^ | -0.65 (-0.76, -0.54)^‡^ | -0.65 (-0.80, -0.49)^‡^ |
| > 1 year - 2 years | -0.75 (-0.89, -0.62)^‡^ | -0.75 (-0.86, -0.64)^‡^ | -0.71 (-0.87, -0.54)^‡^ |
| > 2 years - 3 years | -0.65 (-0.78, -0.51)^‡^ | -0.65 (-0.78, -0.52)^‡^ | -0.61 (-0.78, -0.44)^‡^ |
| > 3 years | -0.31 (-0.44, -0.17)^‡^ | -0.31 (-0.41, -0.20)^‡^ | -0.29 (-0.46, -0.13)^‡^ |
| **Prevalent Geriatric Syndromes** |  |  |  |
| Balance   impairment | 5.69 (5.51, 5.87)^‡^ | 5.72 (5.62, 5.81)^‡^ | 5.35 (5.12, 5.59)^‡^ |
| Bowel   incontinence | 4.53 (4.38, 4.68)^‡^ | 4.52 (4.43, 4.61)^‡^ | 4.61 (4.43, 4.78)^‡^ |
| Cognitive status |  |  |  |
| Intact/borderline | Reference | Reference | Reference |
| Mild/moderate   impairment | 1.67 (1.55, 1.79)^‡^ | 1.67 (1.57, 1.77)^‡^ | 1.68 (1.53, 1.83)^‡^ |
| Moderate-   severe/severe   impairment | 5.27 (5.10, 5.44)^‡^ | 5.26 (5.14, 5.39)^‡^ | 5.33 (5.13, 5.53)^‡^ |
| Hearing   impairment |  |  |  |
| None | Reference | Reference | Reference |
| Hearing   impaired | 0.03 (-0.08, 0.14) | 0.03 (-0.07, 0.14) | -0.02 (-0.14, 0.10) |
| Missing data | 0.66 (-0.15, 1.46) | 0.66 (-0.14, 1.46) | 0.75 (-0.18, 1.68) |
| Body mass index   (BMI) |  |  |  |
| BMI < 18.5 | Reference | Reference | Reference |
| 18.5 ≤ BMI ≤   25 | -0.54 (-0.68, -0.40)^‡^ | -0.54 (-0.67, -0.41)^‡^ | -0.57 (-0.71, -0.42)^‡^ |
| 25 < BMI <30 | -0.87 (-1.03, -0.72)^‡^ | -0.87 (-1.01, -0.73)^‡^ | -0.97 (-1.14, -0.81)^‡^ |
| BMI ≥ 30 | -0.59 (-0.75, -0.43)^‡^ | -0.58 (-0.73, -0.44)^‡^ | -0.72 (-0.91, -0.53)^‡^ |
| Pain |  |  |  |
| None | Reference | Reference | Reference |
| Less than daily   pain | 0.29 (0.19, 0.39)^‡^ | 0.30 (0.21, 0.39)^‡^ | 0.12 (-0.02, 0.26) |
| Daily or severe   daily pain | 0.82 (0.70, 0.94)^‡^ | 0.84 (0.73, 0.94)^‡^ | 0.56 (0.38, 0.75)^‡^ |
| Pressure ulcer | 2.67 (2.52, 2.82)^‡^ | 2.67 (2.52, 2.82)^‡^ | 2.72 (2.56, 2.88)^‡^ |
| Urinary   incontinence | 4.19 (4.04, 4.35)^‡^ | 4.19 (4.10, 4.28)^‡^ | 4.22 (4.05, 4.40)^‡^ |
| Visual impairment |  |  |  |
| None | Reference | Reference | Reference |
| Moderate   impairment | 0.68 (0.59, 0.77)^‡^ | 0.68 (0.60, 0.76)^‡^ | 0.73 (0.62, 0.85)^‡^ |
| Severe impairment | 2.49 (2.33, 2.65)^‡^ | 2.50 (2.36, 2.64)^‡^ | 2.40 (2.22, 2.57)^‡^ |
| **Prevalent Chronic Conditions** |  |  |  |
| Arthritis | 0.08 (-.0003, 0.15) | 0.08 (0.0003, 0.15)^*^ | 0.08 (-0.01, 0.17) |
| Asthma | 0.10 (-0.04, 0.24) | 0.09 (-0.04, 0.23) | 0.20 (0.04, 0.35)^*^ |
| Cancer | -0.12 (-0.19, -0.04)^†^ | -0.11 (-0.19, -0.03)^†^ | -0.18 (-0.27, -0.10)^‡^ |
| Chronic kidney   disease | 0.31 (0.22, 0.40)^‡^ | 0.31 (0.22, 0.39)^‡^ | 0.35 (0.24, 0.45)^‡^ |
| Coronary artery   disease | -0.13 (-0.21, -0.05)^†^ | -0.13 (-0.20, -0.05)^†^ | -0.17 (-0.28, -0.07)**^†^** |
| Chronic   obstructive   pulmonary  disease | -0.07 ( -0.17, 0.02) | -0.07 (-0.16, 0.02) | -0.09 (-0.20, 0.01) |
| Dementia | -0.22 (-0.35, -0.10)^†^ | -0.23 (-0.34, -0.12)^‡^ | -0.21 (-0.39, -0.03)^*^ |
| Diabetes | -0.06 (-0.14, 0.02) | -0.06 (-0.14, 0.01) | 0.06 (-0.04, 0.16) |
| Epilepsy | 0.47 (0.32, 0.61)^‡^ | 0.47 (0.33, 0.61)^‡^ | 0.47 (0.32, 0.62)^‡^ |
| Heart failure | 0.36 (0.27, 0.46)^‡^ | 0.36 (0.27, 0.45)^‡^ | 0.38 (0.28, 0.49)^‡^ |
| Limb paralysis or   amputation | 1.78 (1.63, 1.93)^‡^ | 1.77 (1.63, 1.90)^‡^ | 2.00 (1.84, 2.16)^‡^ |
| Mood disorder | 0.30 (0.22, 0.38)^‡^ | 0.30 (0.22, 0.37)^‡^ | 0.35 (0.26, 0.45)^‡^ |
| Parkinson’s   disease | 1.75 (1.63, 1.87)^‡^ | 1.74 (1.62, 1.87)^‡^ | 1.82 (1.69, 1.94)^‡^ |
| Peripheral   vascular   disease | 0.03 (-0.10, 0.16) | 0.04 (-0.09, 0.16) | -0.03 (-0.18, 0.12) |
| Psychiatric   conditions other   than depression   and dementia | -0.42 (-0.50, -0.33)^‡^ | -0.42 (-0.50, -0.33)^‡^ | -0.39 (-0.49, -0.29)^‡^ |
| Stroke | 0.46 (0.38, 0.55)^‡^ | 0.47 (0.39, 0.55)^‡^ | 0.46 (0.36, 0.55)^‡^ |
| **Random Effects** |  |  |  |
| √ψ | 1.58 | 1.66 | N/A |
| √θ | 4.90 | 4.90 | N/A |
| **Residual Variance** | N/A | N/A | 5.16 |
| **Derived Estimates** |  |  |  |
| R^2^ | 0.627 | 0.624 | 0.626 |
| ρ | 0.095 | 0.103 | N/A |

Legend:

**Model 1:** Adjusted for resident age, sex, marital status, pre-admission neighborhood income quintile, number of days since admission to long-term care home; includes random intercept for long-term care homes

**Reference**: Variable category is the reference group for all other categories within that variable.

**^*^**p-value <0.05
**^†^**p-value <0.01

**^‡^**p-value <0.0001
***√ψ***: Square root of between-long-term care home variance

***√θ***: Square root of within-long-term care home variance

The **null model** of disability containing only random LTCH intercepts and no explanatory resident or LTCH variables had a within-LTCH variance of 66.91 and a between-LTCH variance of 4.16; variances from all multivariable models were compared to these values to estimate proportion of variance explained (*R^2^*).
***R^2^***: The proportional reduction in the estimated total residual variance compared to the null model (Model 1)

**ρ**: Proportion of variance that is explained by LTCH characteristics = ψ/(ψ+θ)

## Table S9

*Sensitivity of Model 1 Findings to Coding of Chronic Conditions*

| **Variables** | **Model 1** | **Model 1 with Chronic Conditions Coded Using Health Administrative Claims Only** | **Model 1 with Chronic Conditions Coded Using RAI-MDS Data Only** |
| --- | --- | --- | --- |
|  | Estimate (95% CI) | Estimate (95% CI) | Estimate (95% CI) |
| **Resident Characteristics** |  |  |  |
| Age |  |  |  |
| 65 – 74 | Reference | Reference | Reference |
| 75 – 84 | 0.04 (-0.08, 0.17) | -0.07 (-0.19, 0.05) | 0.08 (-0.04, 0.21) |
| 85 – 94 | 0.18 (0.04, 0.31)^*^ | -0.01 (-0.15, 0.12) | 0.23 (0.09, 0.36)^†^ |
| 95+ | 0.61 (0.42, 0.80)^‡^ | 0.36 (0.17, 0.54)^‡^ | 0.64 (0.46, 0.83)^‡^ |
| Sex |  |  |  |
| Female | Reference | Reference | Reference |
| Male | -0.37 (-0.46, -0.28)^‡^ | -0.32 (-0.41, -0.23)^‡^ | -0.37 (-0.46, -0.28)^‡^ |
| Marital Status |  |  |  |
| Married | Reference | Reference | Reference |
| Widowed | -0.41 (-0.50, -0.31)^‡^ | -0.42 (-0.51, -0.32)^‡^ | -0.41 (-0.51, -0.32)^‡^ |
| Never married,   separated or   divorced | -0.60 (-0.73, -0.48)^‡^ | -0.63 (-0.75, -0.50)^‡^ | -0.63 (-0.76, -0.51)^‡^ |
| Missing data on   marital status | -0.64 (-0.96, -0.32)^‡^ | -0.67 (-1.00, -0.35)^‡^ | -0.67 (-0.99, -0.34)^‡^ |
| Pre-LTCH Neighborhood Income Quintile |  |  |  |
| 1 (low) | Reference | Reference | Reference |
| 2 | 0.13 (0.01, 0.26)^*^ | 0.14 (0.01, 0.26)^*^ | 0.15 (0.02, 0.27)^*^ |
| 3 | 0.19 (0.07, 0.31)^†^ | 0.20 (0.08, 0.32)^†^ | 0.20 (0.09, 0.32)^†^ |
| 4 | 0.29 (0.17, 0.41)^‡^ | 0.30 (0.17, 0.42)^‡^ | 0.30 (0.18, 0.42)^‡^ |
| 5 (high) | 0.23 (0.10, 0.37)^†^ | 0.25 (0.11, 0.38)^‡^ | 0.24 (0.11, 0.38)^‡^ |
| Missing data | 0.29 (0.14, 0.44)^‡^ | 0.32 (0.17, 0.47)^‡^ | 0.30 (0.15, 0.46)^‡^ |
| Days in LTC Prior to Index Date | - |  |  |
| 0 - 4 months | Reference | Reference | Reference |
| > 4 months - 12   months | -0.65 (-0.78, -0.52)^‡^ | -0.60 (-0.73, -0.47)^‡^ | -0.65 (-0.78, -0.52)^‡^ |
| > 1 year - 2 years | -0.75 (-0.89, -0.62)^‡^ | -0.69 (-0.82, -0.55)^‡^ | -0.78 (-0.91, -0.64)^‡^ |
| > 2 years - 3 years | -0.65 (-0.78, -0.51)^‡^ | -0.57 (-0.71, -0.43)^‡^ | -0.68 (-0.82, -0.54)^‡^ |
| > 3 years | -0.31 (-0.44, -0.17)^‡^ | -0.13 (-0.27, 0.005) | -0.36 (-0.50, -0.23)^‡^ |
| **Prevalent Geriatric Syndromes** |  |  |  |
| Balance impairment | 5.69 (5.51, 5.87)^‡^ | 5.83 (5.65, 6.01)^‡^ | 5.70 (5.51, 5.88)^‡^ |
| Bowel incontinence | 4.53 (4.38, 4.68)^‡^ | 4.60 (4.44, 4.75)^‡^ | 4.55 (4.40, 4.71)^‡^ |
| Cognitive status |  |  |  |
| Intact/borderline | Reference | Reference | Reference |
| Mild/moderate   impairment | 1.67 (1.55, 1.79)^‡^ | 1.64 (1.52, 1.76)^‡^ | 1.77 (1.64, 1.89)^‡^ |
| Moderate-   severe/severe   impairment | 5.27 (5.10, 5.44)^‡^ | 5.20 (5.03, 5.37)^‡^ | 5.41 (5.23, 5.58)^‡^ |
| Hearing impairment |  |  |  |
| None | Reference | Reference | Reference |
| Hearing impaired | 0.03 (-0.08, 0.14) | 0.007 (-0.10, 0.12) | 0.02 (-0.08, 0.13) |
| Missing data | 0.66 (-0.15, 1.46) | 0.60 (-0.20, 1.40) | 0.70 (-0.12, 1.51) |
| Body mass index   (BMI) |  |  |  |
| BMI < 18.5 | Reference | Reference | Reference |
| 18.5 ≤ BMI ≤   25 | -0.54 (-0.68, -0.40)^‡^ | -0.53 (-0.67, -0.39)^‡^ | -0.53 (-0.67, -0.39)^‡^ |
| 25 < BMI <30 | -0.87 (-1.03, -0.72)^‡^ | -0.84 (-0.99, -0.69)^‡^ | -0.86 (-1.01, -0.71)^‡^ |
| BMI ≥ 30 | -0.59 (-0.75, -0.43)^‡^ | -0.56 (-0.72, -0.40)^‡^ | -0.58 (-0.74, -0.42)^‡^ |
| Pain |  |  |  |
| None | Reference | Reference | Reference |
| Less than daily   pain | 0.29 (0.19, 0.39)^‡^ | 0.31 (0.21, 0.41)^‡^ | 0.28 (0.18, 0.37)^‡^ |
| Daily or severe   daily pain | 0.82 (0.70, 0.94)^‡^ | 0.84 (0.72, 0.97)^‡^ | 0.79 (0.67, 0.91)^‡^ |
| Pressure ulcer | 2.67 (2.52, 2.82)^‡^ | 2.75 (2.60, 2.90)^‡^ | 2.67 (2.52, 2.82)^‡^ |
| Urinary   incontinence | 4.19 (4.04, 4.35)^‡^ | 4.23 (4.07, 4.39)^‡^ | 4.21 (4.05, 4.37)^‡^ |
| Visual impairment |  |  |  |
| None | Reference | Reference | Reference |
| Moderate   impairment | 0.68 (0.59, 0.77)^‡^ | 0.70 (0.61, 0.78)^‡^ | 0.68 (0.59, 0.77)^‡^ |
| Severe impairment | 2.49 (2.33, 2.65)^‡^ | 2.48 (2.32, 2.64)^‡^ | 2.49 (2.32, 2.64)^‡^ |
| **Prevalent Chronic Conditions** |  |  |  |
| Arthritis | 0.08 (-.0003, 0.15) | 0.05 (-0.03, 0.12) | 0.10 (0.02, 0.17)^*^ |
| Asthma | 0.10 (-0.04, 0.24) | 0.06 (-0.12, 0.24) | 0.10 (-0.07, 0.28) |
| Cancer | -0.12 (-0.19, -0.04)^†^ | -0.14 (-0.22, -0.05)^†^ | -0.02 (-0.13, 0.09) |
| Chronic kidney   disease | 0.31 (0.22, 0.40)^‡^ | 0.36 (0.26, 0.46)^‡^ | 0.23 (0.11, 0.35)^‡^ |
| Coronary artery   disease | -0.13 (-0.21, -0.05)^†^ | -0.08 (-0.17, 0.0008) | -0.11 (-0.21, -0.01)^*^ |
| Chronic   obstructive   pulmonary  disease | -0.07 ( -0.17, 0.02) | 0.02 (-0.09, 0.13) | -0.07 (-0.17, 0.03) |
| Dementia | -0.22 (-0.35, -0.10)^†^ | -0.21 (-0.33, -0.10)^‡^ | -0.40 (-0.50, -0.30)^‡^ |
| Diabetes | -0.06 (-0.14, 0.02) | -0.01 (-0.10, 0.07) | -0.02 (-0.10, 0.06) |
| Epilepsy | 0.47 (0.32, 0.61)^‡^ | 0.38 (0.19, 0.57)^‡^ | 0.55 (0.38, 0.71)^‡^ |
| Heart failure | 0.36 (0.27, 0.46)^‡^ | 0.36 (0.27, 0.46)^‡^ | 0.29 (0.17, 0.40)^‡^ |
| Limb paralysis or   amputation | 1.78 (1.63, 1.93)^‡^ | -0.07 (-0.21, 0.06) | 1.75 (1.60, 1.91)^‡^ |
| Mood disorder | 0.30 (0.22, 0.38)^‡^ | -0.02 (-0.12, 0.09) | 0.31 (0.23, 0.39)^‡^ |
| Parkinson’s   disease | 1.75 (1.63, 1.87)^‡^ | 1.75 (1.61, 1.88)^‡^ | 1.82 (1.69, 1.95)^‡^ |
| Peripheral   vascular   disease | 0.03 (-0.10, 0.16) | -0.11 (-0.33, 0.11) | 0.08 (-0.06, 0.23) |
| Psychiatric   conditions other   than depression   and dementia | -0.42 (-0.50, -0.33)^‡^ | -0.39 (-0.49, -0.29)^‡^ | -0.45 (-0.57, -0.33)^‡^ |
| Stroke | 0.46 (0.38, 0.55)^‡^ | 0.98 (0.88, 1.07)^‡^ | 0.40 (0.31, 0.49)^‡^ |
| **Random Effects** |  |  |  |
| √ψ | 1.58 | 1.61 | 1.59 |
| √θ | 4.90 | 4.93 | 4.90 |
| **Derived Estimates** |  |  |  |
| R^2^ | 0.627 | 0.622 | 0.626 |
| ρ | 0.095 | 0.053 | 0.095 |

Legend:

**Model 1:** Adjusted for resident age, sex, marital status, pre-admission neighborhood income quintile, number of days since admission to long-term care home; includes random intercept for long-term care homes

**Reference**: Variable category is the reference group for all other categories within that variable.

**^*^**p-value <0.05
**^†^**p-value <0.01

**^‡^**p-value <0.0001
***√ψ***: Square root of between-long-term care home variance

***√θ***: Square root of within-long-term care home variance

The **null model** of disability containing only random LTCH intercepts and no explanatory resident or LTCH variables had a within-LTCH variance of 66.91 and a between-LTCH variance of 4.16; variances from all multivariable models were compared to these values to estimate proportion of variance explained (*R^2^*).
***R^2^***: The proportional reduction in the estimated total residual variance compared to the null model (Model 1)

**ρ**: Proportion of variance that is explained by LTCH characteristics = ψ/(ψ+θ)

## Table S10

*Sensitivity of Model 2 Findings to Exclusion of Admission Assessments*

|  | **Model 2**^\|\|^  (n = 77, 165) | **Model 2 in Sample Excluding Admission Assessments**  (n = 67,863) |
| --- | --- | --- |
|  | Estimate (95% CI) | Estimate (95% CI) |
| Constant | 2.70 (2.18, 3.22)^‡^ | 2.65 (2.10, 3.20) |
| **Resident Characteristics** |  |  |
| Age |  |  |
| 65 – 74 | Reference | Reference |
| 75 – 84 | 0.04 (-0.08, 0.16) | 0.03 (-0.10, 0.16) |
| 85 – 94 | 0.18 (0.04, 0.31)^*^ | 0.15 (0.01, 0.29)^*^ |
| 95+ | 0.61 (0.42, 0.79)^‡^ | 0.60 (0.41, 0.80)^‡^ |
| Sex |  |  |
| Female | Reference | Reference |
| Male | -0.36 (-0.46, -0.27)^‡^ | -0.36 (-0.46, -0.27)^‡^ |
| Marital Status |  |  |
| Married | Reference | Reference |
| Widowed | -0.41 (-0.50, -0.31)^‡^ | -0.43 (-0.53, -0.33)^‡^ |
| Never married, separated or   divorced | -0.60 (-0.73, -0.48)^‡^ | -0.60 (-0.73, -0.47)^‡^ |
| Missing data on marital status | -0.64 (-0.97, -0.32)^‡^ | -0.60 (-0.94, -0.25)^†^ |
| Pre-LTCH Neighborhood Income Quintile |  |  |
| 1 (low) | Reference | Reference |
| 2 | 0.12 (0.002, 0.25)^*^ | 0.08 (-0.04, 0.21) |
| 3 | 0.18 (0.06, 0.30)^†^ | 0.17 (0.05, 0.29)^†^ |
| 4 | 0.27 (0.15, 0.39)^‡^ | 0.29 (0.16, 0.41)^‡^ |
| 5 (high) | 0.23 (0.09, 0.36)^†^ | 0.19 (0.05, 0.34)^†^ |
| Missing data | 0.28 (0.13, 0.43)^‡^ | 0.25 (0.10, 0.40)^†^ |
| Days in LTCH Prior to Index Date | - |  |
| 0 - 4 months | Reference | Reference |
| > 4 months - 12 months | -0.65 (-0.78, -0.52)^‡^ | -0.69 (-0.84, -0.55)^‡^ |
| > 1 year - 2 years | -0.76 (-0.89, -0.62)^‡^ | -0.82 (-0.97, -0.67)^‡^ |
| > 2 years - 3 years | -0.65 (-0.78, -0.51)^‡^ | -0.72 (-0.88, -0.57)^‡^ |
| > 3 years | -0.30 (-0.44, -0.17)^‡^ | -0.41 (-0.55, -0.27)^‡^ |
| **Prevalent Geriatric Syndromes** |  |  |
| Balance impairment | 5.66 (5.48, 5.84)^‡^ | 5.67 (5.48, 5.86)^‡^ |
| Bowel incontinence | 4.52 (4.37, 4.67)^‡^ | 4.57 (4.41, 4.73)^‡^ |
| Cognitive status |  |  |
| Intact/borderline | Reference | Reference |
| Mild/moderate impairment | 1.66 (1.54, 1.78)^‡^ | 1.80 (1.66, 1.93)^‡^ |
| Moderate-severe/severe   impairment | 5.26 (5.09, 5.43)^‡^ | 5.42 (5.24, 5.60)^‡^ |
| Hearing impairment |  |  |
| None | Reference | Reference |
| Hearing impaired | 0.03 (-0.08, 0.13) | -0.02 (-0.13, 0.10) |
| Missing data | 0.67 (-0.11, 1.46) | 0.61 (-0.19, 1.41) |
| Body mass index (BMI) |  |  |
| BMI < 18.5 | Reference | Reference |
| 18.5 ≤ BMI ≤ 25 | -0.54 (-0.68, -0.40)^‡^ | -0.59 (-0.74, -0.45)^‡^ |
| 25 < BMI <30 | -0.88 (-1.03, -0.72)^‡^ | -0.88 (-1.04, -0.72)^‡^ |
| BMI ≥ 30 | -0.60 (-0.76, -0.44)^‡^ | -0.62 (-0.79, -0.45)^‡^ |
| Pain |  |  |
| None | Reference | Reference |
| Less than daily pain | 0.29 (0.19, 0.39)^‡^ | 0.26 (0.16, 0.37)^‡^ |
| Daily or severe daily pain | 0.83 (0.70, 0.95)^‡^ | 0.75 (0.62, 0.88)^‡^ |
| Pressure ulcer | 2.67 (2.52, 2.82)^‡^ | 2.59 (2.44, 2.75)^‡^ |
| Urinary incontinence | 4.20 (4.04, 4.35)^‡^ | 4.39 (4.22, 4.55)^‡^ |
| Visual impairment |  |  |
| None | Reference | Reference |
| Moderate impairment | 0.68 (0.59, 0.76)^‡^ | 0.64 (0.52, 0.73)^‡^ |
| Severe impairment | 2.49 (2.33, 2.65)^‡^ | 2.47 (2.31, 2.63)^‡^ |
| **Prevalent Chronic Conditions** |  |  |
| Arthritis | 0.08 (0.0003, 0.15)^*^ | 0.07 (-0.01, 0.15) |
| Asthma | 0.10 (-0.04, 0.24) | 0.12 (-0.03, 0.26) |
| Cancer | -0.12 (-0.19, -0.04)^†^ | -0.12 (-0.20, -0.03)^†^ |
| Coronary artery disease | -0.13 (-0.21, -0.05)^†^ | -0.14 (-0.23, -0.06)^†^ |
| Chronic obstructive pulmonary   disease | -0.07 (-0.17, 0.02) | -0.09 (-0.19, 0.01) |
| Dementia | -0.23 (-0.36, -0.11)^‡^ | -0.17 (-0.31, -0.04)^†^ |
| Diabetes | -0.06 (-0.14, 0.02) | -0.06 (-0.14, 0.02) |
| Epilepsy | 0.47 (0.33, 0.62)^‡^ | 0.48 (0.33, 0.63)^‡^ |
| Heart failure | 0.36 (0.27, 0.45)^‡^ | 0.35 (0.25, 0.44)^‡^ |
| Kidney disease | 0.31 (0.22, 0.40)^‡^ | 0.25 (0.15, 0.35)^‡^ |
| Limb paralysis or amputation | 1.77 (1.62, 1.92)^‡^ | 1.75 (1.60, 1.91)^‡^ |
| Mood disorder | 0.30 (0.22, 0.38)^‡^ | 0.27 (0.19, 0.35)^‡^ |
| Parkinson’s disease | 1.75 (1.63, 1.87)^‡^ | 1.71 (1.58, 1.84)^‡^ |
| Peripheral vascular disease | 0.03 (-0.10, 0.16) | 0.05 (-0.09, 0.19) |
| Psychiatric conditions other   than depression and dementia | -0.42 (-0.50, -0.33)^‡^ | -0.42 (-0.51, -0.33)^‡^ |
| Stroke | 0.46 (0.38, 0.55)^‡^ | 0.43 (0.34, 0.52)^‡^ |
| LTCH Size |  |  |
| Small (≤64) | Reference | Reference |
| Medium (65 – 128) | -0.05 (-0.32, 0.21) | -0.04 (-0.31, 0.23) |
| Large (129 – 192) | 0.08 (-0.24, 0.40) | 0.10 (-0.22, 0.42) |
| Extra-large ( ≥193) | 0.25 (-0.13, 0.63) | 0.25 (-0.13, 0.64) |
| Ownership Status |  |  |
| Not-for-profit | Reference | Reference |
| For-profit | 0.23 (0.006, 0.46)^*^ | 0.16 (-0.06, 0.39) |
| Missing data | 0.44 (-0.47, 1.35) | 0.22 (-0.81, 1.26) |
| LTCH Location |  |  |
| Rural | Reference | Reference |
| Sub-urban | 0.14 (-0.22, 0.49) | 0.21 (-0.16, 0.57) |
| Urban | -0.12 ( -0.41, 0.15) | -0.11 (-0.39, 0.18) |
| Mean % residents received physio- or occupational therapy (Quartiles) |  |  |
| Lowest quartile | Reference | Reference |
| 2^nd^ quartile | 0.17 (-0.13, 0.47) | 0.01 (-0.30, 0.33) |
| 3^rd^ quartile | 0.14 (-0.17, 0.45) | -0.22 (-0.53, 0.10) |
| Highest quartile | -0.05 (-0.35, 0.24) | -0.12 (-0.42, 0.19) |
| Mean % residents restrained (Quartiles) |  |  |
| Lowest quartile | Reference | Reference |
| 2^nd^ quartile | 0.007 (-0.30, 0.32) | 0.21 (-0.10, 0.51) |
| 3^rd^ quartile | -0.23 (-0.54, 0.07) | 0.17 (-0.14, 0.48) |
| Highest quartile | -0.14 (-0.45, 0.16) | -0.04 (-0.34, 0.25) |
| Median Resident ADL in each home (Quartiles) |  |  |
| Lowest quartile | Reference | Reference |
| 2^nd^ quartile | 1.16 (0.87, 1.46) ^‡^ | 1.07 (0.78, 1.37)^‡^ |
| 3^rd^ quartile | 1.62 (1.30, 1.94) ^‡^ | 1.54 (1.22, 1.86)^‡^ |
| Highest quartile | 2.81 (2.50, 3.11) ^‡^ | 2.72 ( 2.41, 3.03)^‡^ |
| **Random Effects** |  |  |
| √ψ | 1.21 (1.13, 1.28) | 1.21 (1.13, 1.29) |
| √θ | 4.90 (4.84, 4.96) | 4.86 (4.80, 4.92) |
| **Derived Estimates** |  |  |
| R^2^ | 0.642 | 0.647 |
| ρ | 0.057 | 0.058 |

Legend:

**Model 2:** Adjusted for resident age, sex, marital status, pre-admission neighborhood income quintile, number of days since admission to long-term care home, as well as the following long-term care home variables: size, ownership type, location, proportion of residents who recently received physiotherapy or occupation therapy, proportion of residents restrained, and median resident disability. Also a random intercept for long-term care homes.

**Reference**: Variable category is the reference group for all other categories within that variable.

**^*^**p-value <0.05
**^†^**p-value <0.01

**^‡^**p-value <0.0001
***√ψ***: Square root of between-long-term care home variance

***√θ***: Square root of within-long-term care home variance

The **null model** of disability containing only random LTCH intercepts and no explanatory resident or LTCH variables had a within-LTCH variance of 66.91 and a between-LTCH variance of 4.16; variances from all multivariable models were compared to these values to estimate proportion of variance explained (*R^2^*).
***R^2^***: The proportional reduction in the estimated total residual variance compared to the null model (Model 1)

**ρ**: Proportion of variance that is explained by LTCH characteristics = ψ/(ψ+θ)

References

1. Verbrugge LM, Jette AM: The disablement process. Soc Sci Med 1994, **38**(1):1-14.
